# Principles of Genome Evolution in the Drosophila melanogaster Species Group 

**DOI:** 10.1371/journal.pbio.0050152

**Published:** 2007-06-05

**Authors:** José M Ranz, Damien Maurin, Yuk S Chan, Marcin von Grotthuss, LaDeana W Hillier, John Roote, Michael Ashburner, Casey M Bergman

**Affiliations:** 1 Department of Genetics, University of Cambridge, Cambridge, United Kingdom; 2 Genome Sequencing Center, Washington University School of Medicine, St. Louis, Missouri, United States of America; Duke University, United States of America

## Abstract

That closely related species often differ by chromosomal inversions was discovered by Sturtevant and Plunkett in 1926. Our knowledge of how these inversions originate is still very limited, although a prevailing view is that they are facilitated by ectopic recombination events between inverted repetitive sequences. The availability of genome sequences of related species now allows us to study in detail the mechanisms that generate interspecific inversions. We have analyzed the breakpoint regions of the 29 inversions that differentiate the chromosomes of Drosophila melanogaster and two closely related species, D. simulans and *D. yakuba,* and reconstructed the molecular events that underlie their origin. Experimental and computational analysis revealed that the breakpoint regions of 59% of the inversions (17/29) are associated with inverted duplications of genes or other nonrepetitive sequences. In only two cases do we find evidence for inverted repetitive sequences in inversion breakpoints. We propose that the presence of inverted duplications associated with inversion breakpoint regions is the result of staggered breaks, either isochromatid or chromatid, and that this, rather than ectopic exchange between inverted repetitive sequences, is the prevalent mechanism for the generation of inversions in the *melanogaster* species group. Outgroup analysis also revealed evidence for widespread breakpoint recycling. Lastly, we have found that expression domains in D. melanogaster may be disrupted in *D. yakuba,* bringing into question their potential adaptive significance.

## Author Summary

The organization of genes on chromosomes changes over evolutionary time. In some organisms, such as fruit flies and mosquitoes, inversions of chromosome regions are widespread. This has been associated with adaptation to environmental pressures and speciation. However, the mechanisms by which inversions are generated at the molecular level are poorly understood. The prevailing view involves the interactions of sequences that are moderately repeated in the genome. Here, we use molecular and computational methods to study 29 inversions that differentiate the chromosomes of three closely related fruit fly species. We find little support for a causal role of repetitive sequences in the origin of inversions and, instead, detect the presence of inverted duplications of ancestrally unique sequences (generally protein-coding genes) in the breakpoint regions of many inversions. This leads us to propose an alternative model in which the generation of inversions is coupled with the generation of duplications of flanking sequences. Additionally, we find evidence for genomic regions that are prone to breakage, being associated with inversions generated independently during the evolution of the ancestors of existing species.

## Abstract 3

Chromosomal inversion breakpoints were compared between three closely related *Drosophila* species. Many are associated with inverted gene duplications, suggesting that the prevalent mechanism for their generation involves staggered breakpoints.

## Introduction

“Eventually the story of the chromosomal mechanisms and its evolution will have to be entirely rewritten in molecular terms” [1].

Over the last century, very detailed studies have been made by cytogeneticists of the intra- and interchromosomal changes that characterize genome evolution in groups as different as mammals (e.g., [2]) and flies (e.g., [3]; see [1,4] for reviews). Chromosome rearrangements are thought to play an important role in reproductive isolation between species [5–7] and in the adaptation of species to their environments [8–10]. These rearrangements may affect fitness by effectively reducing recombination in heterozygotes, thereby preserving co-adapted gene complexes [11,12], or by exerting position effects on loci neighboring breakpoints by modifying gene expression [13]. Only now, with the availability of “complete” genome sequences, can these structural changes in genomes be studied in the molecular detail, as foreseen by Michael White [1] over 30 years ago (e.g., [14–16]).

Genomic sequence data are beginning to reveal a remarkable diversity of patterns of genome rearrangement in different taxa ([17–21]; reviewed in [22]). For example, we see evidence for the recurrent presence of repetitive sequences near breakpoints [23–25] and evidence for the nonrandom distribution of genome breakpoints [16,26,27]. Moreover, there is evidence that large-scale gene expression domains are maintained as syntenic regions, perhaps because of a functional co-dependency of the genes that reside in these domains [20,28,29]. Comparative genomic data allow us to reconstruct the state of ancestral genome arrangements at key phylogenetic nodes [17,30] and to identify genomic regions conserved during the process of adaptation and divergence [31,32].

The genus *Drosophila* has long been a model for cytogenetic studies of genome evolution. Charles Metz's pioneering comparative studies of metaphase karyotypes in the genus [33], combined with subsequent comparative genetic studies, led Muller [34] to conclude that the integrity of chromosome *arms* is largely preserved in the genus *Drosophila,* despite a 2-fold variation in haploid chromosome number (see also [35]). The maintenance of the gene content of chromosomal arms is due to the paucity of inter-arm rearrangements (i.e., pericentric inversions and translocations) ([36,37]; see [38] for why this is so). Sturtevant and Dobzhansky [39] first showed how chromosome inversions can be used to study the evolutionary history of a species group, such as has been shown subsequently in the case of the endemic Hawaiian picture-winged group [3] or in the cactophilic *repleta* species group of the Americas [40]. *Drosophila* is a species-rich genus—about 1,500 species have been described [41]—and has an evolutionary history of perhaps over 120 million years (Myr; Figure S1; [42]). The wealth of information on genome rearrangement in the genus *Drosophila* can now be studied at the molecular level, using the genome sequences of 12 different species of *Drosophila* that are available (http://rana.lbl.gov/drosophila/). Hitherto, the breakpoint regions of ten well-defined inversions have been characterized in Diptera: eight in *Drosophila* [25,43–49], and two in *Anopheles* [50,51]. Here we investigate the genome-wide patterns of rearrangement among three closely related species: *D. melanogaster, D. simulans,* and *D. yakuba.*

*D. melanogaster, D. simulans,* and D. yakuba are all members of the *melanogaster* species subgroup, a collection of nine species of Afrotropical origin [52]. D. melanogaster and D. simulans are cosmopolitan sibling species that split from a common ancestor about 5.4 Myr ago [42] and can form (normally infertile) hybrids. Their polytene chromosome banding patterns are very similar, differing by only one large, and four small, paracentric inversions [53,54]. By contrast, *D. yakuba,* a species of the African savanna, is completely isolated reproductively from D. melanogaster and D. simulans. These three species shared a common ancestor about 12.8 Myr ago [42]. The polytene chromosomes of D. yakuba differ from those of D. melanogaster by at least 28 fixed inversions [54]. The combination of prior cytological knowledge of inversion history and the close evolutionary distance among species in this group provides an unparalleled opportunity to reconstruct the detailed molecular events underlying genome rearrangements between animal genomes.

We studied the first interspecific inversion ever to be documented, *In(3R)84F1;93F6–7,* which differentiates chromosome 3 of D. melanogaster and the species of the *simulans* clade [55,56]. We characterized its breakpoint regions at the molecular level, i.e., the genomic regions that encompass both the sites of chromosome breakage and adjacent sequences. We detected inverted duplications of sequences present in the breakpoint regions, a pattern also shown by the breakpoint regions of other chromosomal rearrangements recently characterized [49,51,57]. One of the breakpoint regions associated with this inversion overlaps that of another inversion that took place on the lineage to *D. yakuba,* suggesting that some genomic regions are repeatedly broken over time. By a large-scale comparison of the molecular organization of the genomes of D. melanogaster and *D. yakuba,* we asked if the features associated with inversion *In(3R)84F1;93F6–7* reflect a recurrent pattern of genome rearrangement in the *melanogaster* species subgroup.

We found that approximately 59% (17/29) of the inversions fixed between D. melanogaster and D. yakuba show evidence of inverted duplication of protein-coding genes or other nonrepetitive sequences present at the breakpoint regions. The prevalence of inverted duplications at inversion breakpoint regions suggests a mechanism of staggered breaks, either isochromatid or chromatid, as the most parsimonious explanation for their origin. Computational analyses failed to find support for the generalized presence of dispersed, repetitive sequences in co-occurrent breakpoint regions, i.e., those that set the limits of a particular inversion. We conclude that the generation of chromosomal rearrangements in the lineages studied is not necessarily linked to ectopic recombination events between repetitive sequences. We also find evidence for the independent breakage of the same genomic region in different lineages, i.e., fragile regions [16,25–27], and in one case, we are able, for the first time in Diptera, to reconstruct the reuse of a breakpoint region.

## Results/Discussion

### Experimental and Computational Analysis of Inversion *In(3R)84F1;93F6–7* Fixed between D. melanogaster and Species of the *simulans* Clade

**Figure 1:**
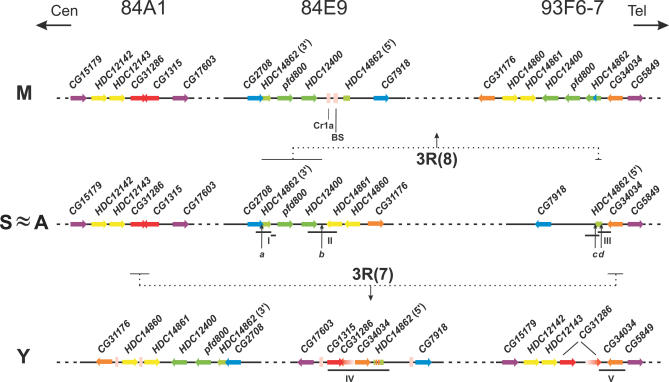
Molecular Organization of Three Genomic Regions of the Right Arm of Chromosome 3 in *D. melanogaster, D. yakuba,* and in D. simulans These three genomic regions harbor the breakpoints of the paracentric inversions 3R(7) and 3R(8), also known as *In(3R)84F1;93F6–7,* and have been reconstructed by BLAST analysis, in situ hybridization, resequencing, and whole-genome alignments at UCSC (http://genome.ucsc.edu/). According to the information in D. erecta and different outgroup species (Table 1), D. simulans (S) is the species that best represents the ancestral (A) configuration for all three regions. Reference genes at the different breakpoint regions have been colored red, blue, and orange. Between some of the reference genes, putatively expressed genes (green and yellow; [60]) and repetitive sequences (pink) are also present. Other surrounding genes are indicated in brown. Top, cytological coordinates of the regions in D. melanogaster (M). Long horizontal lines indicate chromosomes; solid pattern indicates key region; and dashed pattern indicates chromosomal stretch separating key regions. Cen, centromere; Tel, telomere. The head of each colored horizontal arrow represents the 3′ end of each gene or putative gene. Chromosomal segments included in the inversion 3R(7) and 3R(8) are indicated by dotted lines. For both inversions, the sequences between paired staggered breakpoints are indicated by short horizontal solid lines. Roman numerals indicate different chromosomal stretches spanning inversion breakpoints that were sequenced as a control. Vertical arrows indicate the localization in the ancestor *(D. simulans)* of the four breakpoints *(a, b, c,* and *d)* that are necessary to explain the inversion 3R(8) and the duplication of *HDC14862, pfd800,* and *HDC12400* at 84E9 (3R:3862326–3867817; 3R:3874931–3876653) and 93F6–7 (3R:17554739–17562483) of D. melanogaster (see Figure 2). The gene configuration *CG7918-CG34034-CG5849* has been disrupted independently in the lineages of D. melanogaster and D. yakuba (Y) by the inversions 3R(8) and 3R(7), respectively. In *D. melanogaster,* the gene pair *CG2708 (Tom34)-CG31176* is also disrupted, whereas in *D. yakuba, CG31286-CG1315* is disrupted. Inversion 3R(8) and its associated duplication event generate an apparently full copy of the putative expressed gene *HDC14862* in 3R:93E10-F2 of *D. melanogaster.* This contains 56–59 bp from the 3′ UTR of the gene *CG2708* (blue triangle) within one of its putative introns. *HDC14862* is present as two different fragments both in D. simulans and D. yakuba (see main text for details). Further, the inversion 3R(7) has disrupted the antisense overlap of *CG31286* and *CG1315* in *D. yakuba:* the antisense configuration is conserved at 84A1 of D. melanogaster and *D. simulans,* as well as in other species (Table 1). Inversion 3R(7) was accompanied by a duplication of *CG34034* and a complex pattern of rearrangement that also involved a fragment of the 5′ region of *HDC14862*. The two open reading frames (ORFs) of *CG34034* are functional according to GENSCAN (http://genes.mit.edu/GENSCAN.html), although the putative protein sequences they encode differ substantially from that of their orthologs in D. melanogaster and D. erecta. Some stretches with significant homology with *CG31286* are also detected adjacent to *CG1315* in D. yakuba. The reference gene *CG31286* is also tandemly duplicated and adjacent to *CG34034*. In *D. yakuba,* there are three copies of *CG31286,* two of them being pseudogenes (denoted as a red gradient). Only the copy immediately distal to *HDC12143* is functional, although it apparently codes for only one of the two isoforms of its D. melanogaster ortholog. Genes and distances between them are not represented proportionally.

In a remarkable study, Sturtevant and Plunkett [56] deduced from genetic evidence that the chromosomes of D. melanogaster and D. simulans differed by an inversion on the right arm of chromosome 3. This inversion was later confirmed by an analysis of the polytene chromosomes of the interspecific hybrids ([58];see also [53]). We have directly cloned the breakpoints of this inversion from the genome of D. simulans and, by a combination of experimental and computational methods, characterized the breakpoint regions in the genome sequences of *D. melanogaster, D. simulans,* and *D. yakuba.* The structure of the two breakpoint regions of this inversion is illustrated in Figure 1.

**Table 1:**
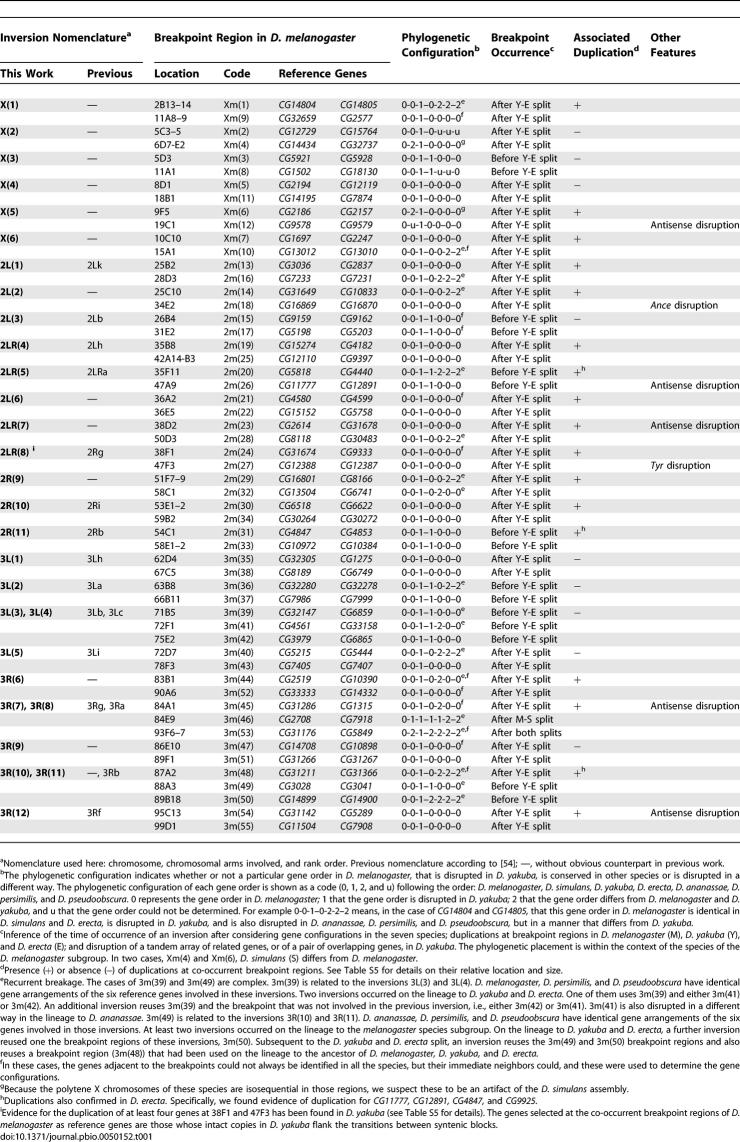
Co-Dependent Breakpoint Regions in D. melanogaster versus D. yakuba and Inferences on the Phylogenetic Occurrences of Inversions

**Figure 2:**
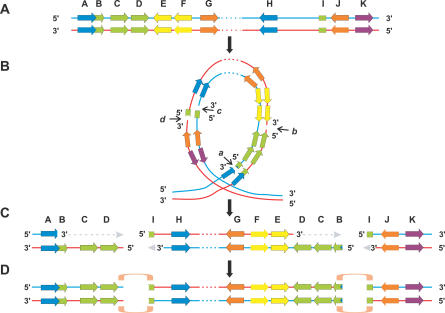
An Isochromatid Model with Staggered Single-Strand Breaks Can Give Rise to an Inversion Accompanied by Duplications at the Breakpoint Regions in Inverted Orientation The mechanism is illustrated in relation to the inversion 3R(8), which is fixed in the lineage to D. melanogaster. (A) Ancestral state in D. simulans (Figure 1). (B) Two pairs of staggered single-strand breaks *(a-b* and *c-d)* result in long 5′-overhangs (C), which can then be filled in (grey dashed arrow); when followed by nonhomologous end joining, this may result in an inversion flanked by inverted duplications of the sequences between the paired single-strand breaks (D). Landmarks: A, *CG2708;* B, *HDC14862* (3′); C, *pfd800;* D, *HDC12400;* E, *HDC14861;* F, *HDC14861;* G, *CG31176;* H, *CG7918;* I, *HDC14862* (5′); J, *CG34034;* and K, *CG5849*. Color code as in Figure 1. Figure S2 illustrates the model for the formation of the inversion 3R(7).

To clone the *In(3R)84F1;93F6–7* breakpoints, we performed in situ hybridizations to polytene chromosomes of D. simulans (and to those of D. melanogaster OR-R as a control), using five D. melanogaster bacterial artificial chromosomes (BACs) that we expected to cross the breakpoints of the major D. simulans inversion at 84F1 (BACR07M14 and BACR45A07) and at 93F6–7 (BACR16N15, BACR42I20, and BACR08K01) [54]. A BAC that includes an inversion breakpoint must necessarily yield two hybridization signals on chromosome arm 3R of *D. simulans,* but only one on that of D. melanogaster. We determined that BACR07M14 contains the proximal breakpoint and that BACR16N15 contains the distal breakpoint of this inversion. The breakpoints within these BACs were narrowed down by in situ hybridization with probes of genes selected from the predicted cytological coordinates of the breakpoints [54]. We determined that the limits of this inversion were between the protein-coding genes *CG2708* and *CG7918,* proximally, and *CG31176* and *CG34034,* distally.

The gene pairs *CG2708-CG7918* and *CG31176-CG34034* delimit two breakpoint regions in D. melanogaster of 22.6 and 17.8 kilobases (kb) long at 84E9–10 and 93E10-F2, respectively (Figure 1). Neither region contains any annotated protein-coding genes in the *Drosophila* genome Release 4.3 annotation (http://chervil.bio.indiana.edu:7092/annot/), with only the non-LTR retrotransposons *BS* and *Cr1a* in the region at 84E9–10 as identifiable features [59]. We further characterized the inversion breakpoint regions in D. melanogaster by BLAST analysis and found the presence of four putatively expressed sequences [60] and a sequence said to be related to the mammalian proto-oncogene *c-fos (pfd800).* The order of the sequences at these breakpoint regions is, from centromere to telomere: *HDC14862*-*pfd800*-*HDC12400-Cr1a-BS-HDC14862* at 84E9–10, and *HDC14860-HDC14861-HDC12400-pfd800-HDC14862* at 93E10-F2 (Figure 1).

Notably, three of these sequences *(HDC14862, pfd800,* and *HDC12400)* are present at both breakpoint regions in an inverted orientation with respect to each other (Figure 1). The nucleotide identity between duplicated stretches is about 95% across approximately 6.3 kb of aligned sequence. Their divergence is greater than the divergence of the *Cr1a* and *BS* sequences from the consensus sequences of these elements, 3.2% and 0.5%, respectively. This suggests that the transposable elements (TEs) inserted more recently than the duplication event.

The location of the inverted duplicated sequences at both breakpoint regions was confirmed by in situ hybridization. Sequences in this duplicated interval are not found elsewhere in the genome of *D. melanogaster,* as shown both computationally by BLAST analysis and experimentally by in situ hybridization with appropriate probes. Using probes for the *HDC14862, pfd800,* and *HDC12400* sequences, we found that the duplication is also present in the Zimbabwe 2 strain of *D. melanogaster,* which is from an ancestral population relative to cosmopolitan and laboratory strains [61], suggesting the duplication is widespread or fixed in D. melanogaster. Furthermore, BLAST analysis against the D. simulans and D. yakuba genomes suggested (see Materials and Methods), and interspecific in situ hybridization confirmed, that the region duplicated in D. melanogaster is present as a single copy in both the D. simulans and D. yakuba genomes. This analysis indicates that the duplication of sequences associated with the breakpoint regions in D. melanogaster represents the derived state relative to that of D. simulans. A similar pattern of inverted duplicated sequences at breakpoint regions has been reported for the polymorphic inversion *In(3R)P* in D. melanogaster [49], the polymorphic inversion *In(2L)a* in Anopheles gambiae [51], and for the pericentric inversion fixed between Pan troglodytes chromosome 10 and the homologous Homo sapiens chromosome 12 [57].

The comparison of the molecular organization of the breakpoint regions of *In(3R)84F1;93F6–7* between *D. melanogaster, D. simulans,* and the outgroup species D. yakuba revealed that a second inversion fixed in the lineage that leads to D. yakuba reused one of the *In(3R)84F1;93F6–7* breakpoint regions. In *D. yakuba,* the *CG2708-CG31176* breakpoint region is identical in molecular organization to that of *D. simulans,* further supporting the hypothesis that *In(3R)84F1;93F6–7* is derived, occurring on the D. melanogaster lineage. In contrast, the gene *CG7918* remains adjacent to *CG34034,* but in a different chromosomal location from that of *CG5849,* which is in turn adjacent to a second copy of *CG34034*. In *D. simulans, D. erecta,* and other distantly related species (Table 1), the genes *CG7918, CG34034,* and *CG5849* are collinear and *CG34034* is present in a single copy. In *D. yakuba,* the gene pairs *CG7918-CG34034* and *CG34034-CG5849* are found close to the genes *CG1315* and *CG31286,* respectively. *CG1315* and *CG31286* are adjacent in *D. melanogaster, D. simulans,* and other *Drosophila* species (Table 1), indicating this to be the ancestral organization for this region. Therefore, the *CG7918-CG34034-CG5849* interval has been independently disrupted by another inversion on the D. yakuba lineage, although the precise breakpoints differ from those associated with *In(3R)84F1;93F6–7.* This inversion on the D. yakuba lineage is associated with inverted duplications of *CG34034* and *CG31286* (Figure 1; see below). The reuse of the breakpoint region *CG7918*-*CG34034* is the second example in *Drosophila* of recurrent breakage, demonstrated at the molecular level [25], and is the first in which the associated inversion events can be unambiguously deciphered.

The association of inverted duplications with these breakpoint regions is not consistent with a model of inversion origin by recombination between two copies of the same TE [62]. We propose a model of staggered breaks. These breaks may either be isochromatid (Figures 2 and S2, see also [57]), occurring during premeiotic mitosis, or chromatid, occurring during meiotic prophase (Figure S3). A potential difficulty of the isochromatid model is the length of DNA that would need to be unwound, presumably by helicase activity. Alternative mechanisms, such as multiple rearrangements or recombination between two independent, but similar, inversions [38], cannot be ruled out, but they are less parsimonious. In either case, the frequent presence of duplications at co-occurrent breakpoint regions argues against a simple “cut-and paste” mechanism of inversion formation [44]. An important implication of our model is that the presence of inverted duplications at co-occurrent breakpoint regions allows the unambiguous determination of the polarity of chromosome change [49,51]. Traditionally, phylogenetic trees of *Drosophila* based on inversion analysis have been unrooted (e.g., [3,54]). Outgroup analysis can allow the determination of ancestral and derived states, as realized for polytene chromosome inversion phylogenies ([63]; see also [64]), but the widespread signature of inverted duplications provides another independent source of data for polarizing inversion history (see below).

In the case of *In(3R)84F1;93F6–7,* four breaks (*a, b, c,* and *d* in Figure 1) would have occurred in an ancestral chromosomal arrangement that is now best represented in the D. simulans genome. The breakpoint pairs *a-c* and *b-d* (which have been confirmed by resequencing; Figure 1) would each represent staggered breaks within a single chromatid in Figure 2. *CG2708* and *HDC14862* overlap by 56–59 base pairs (bp) in D. simulans. Breakpoint *a* occurred at the 5′ end of this overlap, duplicating this region in D. melanogaster. Breakpoint *b* occurred in the region between *HDC12400* and *HDC14861*. Breakpoint *c* occurred downstream of the “exon” 2 of the distal, partial copy of *HDC14862* in *D. simulans,* which roughly corresponds to the intron between “exons” 2 and 3 of the “complete” copy of *HDC14862* of D. melanogaster (roughly upstream of the start of the overlapping region with *CG2708*). The fourth breakpoint, *d,* is found 1,760–1,764 bp downstream of breakpoint *c* in *D. simulans,* at 25 bp from the start of the “exon” 1 of *HDC14862*. End-filling followed by nonhomologous end joining in the inverted orientation (Figure 2) would result in both the inversion *In(3R)84F1;93F6–7,* the duplication of the region including *HDC14862, pfd800,* and *HDC12400,* and the fortuitous formation of what is considered a “complete” copy of the putatively expressed sequence *HDC14862.*

### Comparative Analysis of Genome Organization between D. melanogaster and D. yakuba

**Figure 3:**
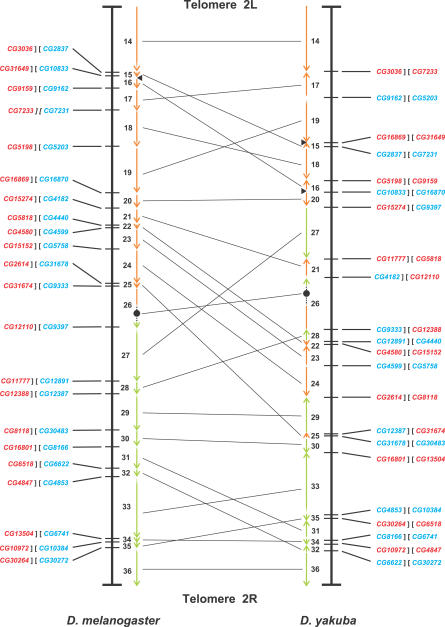
Large-Scale Comparison of the Genomes of D. melanogaster and D. yakuba. Muller's Elements B and C (chromosome 2) Similar plots are shown for Muller's element A (chromosome X) and Muller's elements D and E (chromosome 3) in Figure S4A and S4B, respectively. The outermost protein-coding genes of consecutive syntenic blocks are indicated. Following Bridges [116], syntenic blocks (defined as regions in which the relative gene order is globally conserved between D. melanogaster and D. yakuba) are numbered taking D. melanogaster as a reference and in an increasing order from the telomere of chromosome X (number 1) to the telomere of the right arm of chromosome 3 (number 58); an arrowhead indicates the orientation of the segments. Lines between chromosomes match homologous syntenic blocks between species. The pericentric inversion between Muller's elements B and C during the divergence of D. melanogaster and D. yakuba is shown by a color code, whereas syntenic blocks on the left and right arms of D. melanogaster appear in orange and green, respectively; the syntenic block that contains the centromere, number 26, is not colored. The solid triangles denote the gene *CG6081*, whose duplication accompanied the origin of the inversion 2L(2) (Figures 2, S2, and S3).

We used a computational approach to identify genome-wide disruptions in gene order between the chromosomes of D. melanogaster and *D. yakuba.* Each D. melanogaster transcript was used as a query in a high stringency (E < 10^−30^) BLASTN search against the genomic sequence of D. yakuba. This allowed us to map unambiguously 12,690 genes (94.4% of those of Release 4.1) of D. melanogaster on the genome sequence of D. yakuba. A comparison of the gene orders of the two species identified 55 gene-order disruptions between them, which appear as discontinuities in the coordinates of neighboring genes in one species relative to the other (Tables 1 and S1). All predicted gene-order disruptions identified using this gene-based BLAST approach are also identified as termini of whole-genome global alignments at the University of California, Santa Cruz (UCSC) [65]. These 55 gene-order disruptions define 59 syntenic blocks between these species (since both species have four chromosomes) (Table S2). The location and relative orientation of the syntenic blocks for chromosome 2 of D. melanogaster and D. yakuba are shown in Figure 3; similar data are shown for chromosomes X and 3 in Figure S4. We do not show the small chromosome 4 (syntenic block 59), since our results indicate that this chromosome is wholly collinear in the two species over the sequenced region [66]. Syntenic blocks 13, 26, and 46 include the centromeric heterochromatic regions for chromosomes X, 2, and 3, respectively. We are unable, given the present sequence data, to detect any chromosome rearrangements within these heterochromatic regions or those on chromosome 4.

To obviate possible artifacts of the assembly process (see Material and Methods) on our results, and directly confirm our predictions of the gene order around the D. yakuba breakpoint regions relative to those of *D. melanogaster,* we cloned and sequence verified a sample of 27 of the predicted breakpoint regions from *D. yakuba,* each containing the transition between adjacent syntenic blocks (see Materials and Methods). In every case, our predictions were directly confirmed (Table S1). This result is consistent with the fact that all predicted gene-order disruptions are found in high-quality, contiguous (i.e., ungapped) regions of the D. yakuba assembly. In fact, breakpoint regions in D. yakuba are sequenced to an average depth of 8× and are supported by an average of 14 clone pairs. These results demonstrate that the gene-order disruptions inferred between the D. yakuba and D. melanogaster genomes are not assembly artifacts.

Approximately 117.8 megabases (Mb) of the D. melanogaster genome and about 118.9 Mb of the D. yakuba genome are included in the 59 syntenic blocks as defined by their outermost markers or reference genes. The amount of nonheterochromatic DNA not included in these syntenic blocks is 542 kb of the D. melanogaster genome and 674 kb of the D. yakuba genome. This is an upper estimate because in some cases, there is noncoding homology between the reference genes that define two consecutive syntenic blocks (see below). The median size of syntenic blocks is 1.66 Mb in *D. melanogaster,* and 1.61 Mb in D. yakuba. Excluding the syntenic blocks that contain centromeric heterochromatin (blocks 13, 26, and 46), the largest (syntenic block 57) is just over 6 Mb (~5.2% of the genome in both species), and the smallest is 161 kb (syntenic block 22, 0.08% of the D. melanogaster genome; and syntenic block 25, 0.08% of the D. yakuba genome). The length of genomic regions in each syntenic block is highly correlated across species (Spearman ρ = 0.997, *p* = 3.78 × 10 ^−61^; blocks 13, 26, and 46 not included), and in only two cases (blocks 26 and 43), do they differ by more than 10%. The DNA content per syntenic block does not differ significantly between D. melanogaster and D. yakuba (Wilcoxon signed rank test, *Z* = −1.273, *p* = not significant [n.s.]; blocks 13, 26, and 46 not included). A departure of the observed distribution of the lengths of syntenic blocks from that expected if the breakpoints were randomly distributed across the genome (a truncated negative exponential distribution) would allow us to discard the random breakage model of chromosome evolution [26,67]. Based on the comparison of the empirical and theoretical distributions, we cannot reject the random breakage model (Kolmogorov-Smirnov test, *D* = 0.2, *p* = n.s.; blocks 13, 26, and 46 not included).

Despite the conservative criteria used in our BLAST analysis, its resolution is sufficient to detect gene sequences that may have “escaped” synteny by transposition, as has been observed in *Drosophila* both experimentally, e.g., [68], and by genomic analyses [69–71]. We detected 22 potential transposition events between D. melanogaster and *D. yakuba,* with 12 occurring unambiguously *between* chromosome arms and eight events *within* chromosome arms (Tables S3 and S4). This number is likely to be an underestimate because we used stringent criteria for paralogy. Of the 22 events that we detected, 20 are duplicative transpositions and two are conservative transpositions.

### Reconstruction of the Inversion History between D. melanogaster and D. yakuba

Muller [34] defined the six fundamental elements of the karyotype of the genus *Drosophila* (now referred to as Muller's elements A–F, each corresponding to a chromosome arm of D. melanogaster). The overall gene content of these elements has been conserved during the evolution of the genus as witnessed by the very few inter-element rearrangements (i.e., pericentric inversions and translocations) that have been reported. Previous analysis of inversion differences between D. melanogaster and D. yakuba based on polytene chromosome revealed 28 inversions, of which only one, on chromosome 2 was pericentric [54] (Table 2).

**Table 2:**
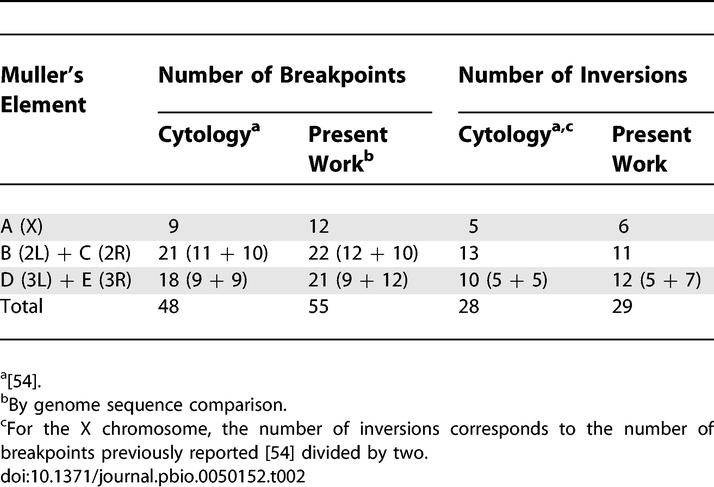
Magnitude of Chromosomal Change between D. melanogaster and D. yakuba for the Different Muller's Chromosomal Elements

We established which pairs of breakpoint regions define particular inversions by taking into account the contiguity relationships in both species of the outermost genes of syntenic blocks between D. melanogaster and D. yakuba (Figures 3 and S4; Table S1). In general, our computational analysis of the genome sequences of these two species is broadly compatible with previous results based on polytene chromosomes [54]. We inferred that 29 inversions distinguish the chromosomes of D. melanogaster and *D. yakuba,* of which 28 are paracentric and one corresponds to the pericentric inversion on chromosome 2 (Table 2). The total number of inversions inferred computationally is just one more than that suggested by polytene chromosome analysis [54], although the greater resolution of the sequence analysis increases the number of breakpoints from 48 to 55 and refines their positions (Tables 1 and 2).

Our analysis shows many discrepancies in detail when compared to previous work ([54]; Tables 1 and 2). This is especially true on the X chromosome, where the banding pattern has diverged greatly in the *melanogaster* species group. On chromosome 2, there is what Lemeunier and Ashburner [54] interpreted as a single pericentric inversion, which distinguishes D. yakuba and its relatives, *D. teissieri, D. erecta,* and *D. orena,* from D. melanogaster and the three species of the D. simulans clade. As shown in Figure 3, there is a complex mosaic of syntenic blocks between the two arms of chromosome 2. In good agreement with the previous work [54], a single pericentric inversion, 2LR(5), is sufficient to explain this pattern. This inversion has identical limits in both D. yakuba and D. erecta. Inverted duplications at the breakpoint regions in both species (Table S5, see below) and information on gene order in other outgroup species (Table 1) strongly suggest that this inversion occurred in the common ancestor of D. yakuba and D. erecta after this lineage split from that leading to the *melanogaster-simulans* complex. Figure S6 illustrates one of the most parsimonious scenarios that explains the evolution of chromosome 2.

### Inversion-Mediated Duplication Is Frequent at Breakpoint Regions

We characterized in detail the sequences of the 55 breakpoint regions of D. yakuba because genomic and phylogenetic evidence suggested that virtually all inversion events between D. melanogaster and D. yakuba occurred on the D. yakuba lineage (Table 1; see below). Remarkably, in 34 of 55 (approximately 62%) breakpoint regions, we detected the presence of duplications of sequences that are only present once in the genome of *D. melanogaster.* In each case, these duplications are specifically associated with the pair of breakpoint regions that limit a particular inversion (Table S5; see below). These duplications are not repetitive in the D. yakuba genome (by BLAST analysis), nor do they match any identifiable *Drosophila* TE. In a control experiment, the genomic regions of D. melanogaster that correspond to the co-occurrent breakpoint regions of D. yakuba were compared to each other. Repetitive sequences were found in six cases; in no case other than that of *In(3R)84F1;93F6–7* (see Figure 1) were duplications of unique sequences found.

In total, 18 of 29 inversions (approximately 62%) fixed between *D. melanogaster* and *D. yakuba* are associated with duplications of sequences included at co-occurrent breakpoint regions. These duplicated sequences are in opposite orientations in the co-occurrent breakpoints of 17 inversions; 3R(6) is the only exception, potentially as a result of a subsequent microinversion [72]. These sequence duplications include 22 full or partial duplications of protein coding genes. Most of these (exceptions are *CG14817* at Xy(1) and Xy(4), *CG6081* at 2y(15) and 2y(18), and *CG34034* at 3y(46) and 3y(53)) have accumulated many point and indel mutations, and are presumed to be nonfunctional. The average nucleotide identity (± the standard deviation [SD]) between duplicates is approximately 88% ± 5.4%. For six of the inversions, sequences from both breakpoint regions are present as inverted duplications at each breakpoint. For the remaining 12 inversions, sequences from only one of the two breakpoint regions are duplicated. This may be due either to the evolutionary loss, by sequence change, of one of the copies of an original duplication, or to the fact that only one of the pair of single-stranded breaks was significantly staggered (Figure S5A and S5B, respectively). The size of the duplications varies significantly in D. yakuba (median = 321 bp, coefficient of variation [CV] = 81% counting only one of the copies when in tandem; Table S5), but in no case do they involve more than about 1.9 kb of aligned sequence (the shortest duplication is 46-bp long).

In many taxa, repeated sequences have been found to be associated with rearrangement breakpoints and have been implicated in mediating chromosomal rearrangements by a process of ectopic exchange. This has been the case for tRNAs and ribosomal protein genes in yeasts [73,74], segmental duplications in the human-mouse [24] and human-primate lineages [75–78], and TEs in many organisms [46,79–81]. In *D. melanogaster,* there is abundant experimental evidence that exchange between TEs can result in chromosome rearrangement (e.g., [82]). Comparative sequence data also indicate that TEs are abundant at interspecific breakpoint regions between Diptera species [25,69], and there is strong evidence implicating TE-mediated ectopic exchange events in four [25,46,47,51] of the ten well-defined inversions whose breakpoint regions have been characterized at the molecular level (Table 3).

**Table 3:**
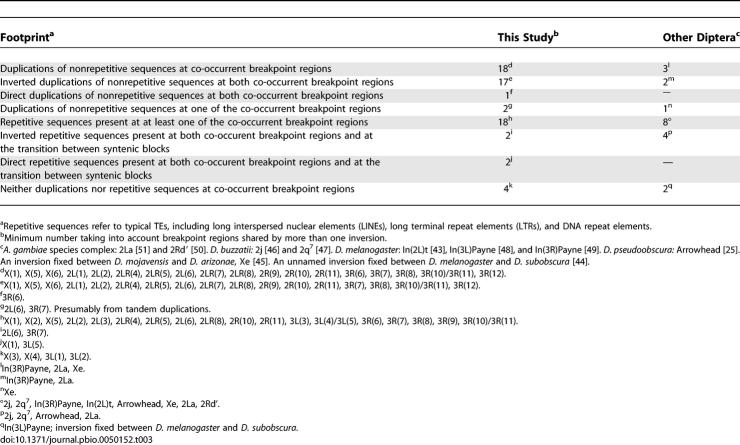
Presence of Duplications and Repetitive Sequences at Breakpoint Regions of Characterized Dipteran Inversions

We analyzed the breakpoint regions of D. yakuba for TE sequences using RepeatMasker with the Release 4.2 TE annotation of the D. melanogaster genome [83] and by BLAST2 analysis using as a query TEs sequences from species other than D. melanogaster. Over 45% of breakpoint regions (25/55) include repetitive sequences in D. yakuba (Table S6), but only five co-occurrent pairs of breakpoint regions (involving inversions 2LR(5), 2L(6), 2LR(8), 3L(3)/3L(4), and 3R(6)) include a similar repetitive sequence (Table S6). These analyses would fail to detect any repetitive sequence absent from the RepeatMasker library (as would be those exclusive to D. yakuba) or not yet characterized in D. yakuba. For this reason, we manually extracted from the D. yakuba breakpoint regions a set of sequences, each corresponding to the precise transition region between syntenic blocks, and used them as BLAST queries to the entire D. yakuba genome. Similar repetitive sequences were found at the co-occurrent breakpoints of the inversions X(1), 2L(6), 3L(5), and 3R(7), although only in the case of 2L(6) and 3R(7) are the copies of the repetitive sequence inverted with respect to each other. The average length of these sequences was 685 bp and the range 49–3,037 bp. Unfortunately, we can neither date the insertion of these repetitive sequences (with respect to the time of occurrence of the inversion), nor can we assert that the *absence* of repetitive sequences at other pairs of co-occurrent breakpoint regions is not due to their decay or loss subsequent to the occurrence of an inversion. Nevertheless, these data provide little direct evidence for the presence of TEs in generating fixed inversions between D. melanogaster and *D. yakuba* and, combined with the recurrent presence of inverted duplications of nonrepetitive sequences, suggests that ectopic recombination between TEs has not been the dominant mechanism of generating inversions in this lineage. These results contrast with the presence of inverted TEs at co-occurrent breakpoints of well-defined inversions (Table 3).

### Lineage-Specific Rates of Chromosomal Evolution

We mapped the derived state of the 29 inversions between the two genomes to the D. melanogaster or D. yakuba lineages, using several independent criteria (Table 1): (1) by determining the arrangement of each gene pair disrupted by an inversion in D. melanogaster versus *D. yakuba* in five other sequenced *Drosophila* species; (2) by the presence of inverted duplications associated with co-occurrent breakpoints, as discussed above; and (3) by the disruption of a tandem array of related genes, or of a pair of genes whose transcripts show 3′-overlap (see below), which we also consider to be a derived state. In all cases in which we can use more than one of these criteria, all are consistent. Our analyses show that of 29 inversions, 28 have been fixed in the lineage leading to *D. yakuba,* and only one (3R(8), also known as *In(3R)84F1;93F6–7*) on the lineage leading to D. melanogaster (eight of the former inversions occurred before the D. erecta/D. yakuba split). This difference is highly significant (one-tailed binomial *p* = 5.59 × 10^−8^) and agrees well with previous interpretations [64], demonstrating that rates of chromosomal evolution can vary by over an order of magnitude even among closely related species. The origin of this very asymmetric rate of fixation cannot stem from differences in the degree of intraspecific polymorphism, as has been proposed for D. pseudoobscura and D. subobscura [84], because D. melanogaster is substantially more polymorphic for inversions than D. yakuba [54]. Rather, it might reflect different effective population sizes between the African populations of the immediate ancestors of D. melanogaster and D. yakuba [85,86].

We used the number of breakpoints per Mb per Myr to correct for differences in chromosomal size in a comparison of rates of chromosomal evolution between species pairs of different *Drosophila* groups (Table 4) in which we assumed a constant rate of evolution as a null hypothesis. In view of the pericentric changes in chromosome 2 (Muller's elements B+C), we combined the data for these elements. The overall rate of breakage in the *D. melanogaster/D. yakuba* lineage is 0.0183/Mb/Myr. This is slower than that seen in the *D. pseudoobscura/D. miranda* (*G*_adj_ = 38.9; *d.f.* = 1; *p* < 4.4 × 10^−10^) and *D. pseudoobscura/D. subobscura* (*G*_adj_ = 48.5; *d.f.* = 1; *p* < 3.4 × 10^−12^) comparisons, comparable with the rate seen in the comparison *D. virilis/D. montana* (*G*_adj_ = 0.5; *d.f.* = 1; *p* = n.s.) and accelerated with respect to that in the *repleta* species group (*G*_adj_ = 4.3; *d.f.* = 1; *p* < 4.3 × 10^−9^). Across Muller's elements, the rank order of the rate of chromosome evolution is A > (B+C) > E > D, which agrees well with the genus-wide pattern of rates of evolution A > E > D proposed by [87], based on the comparisons of D. melanogaster and D. repleta [21,87] and of *D. virilis, D. montana,* and D. novamexicana [88]. Nevertheless, Muller's elements B+C appear to have evolved faster in the *D. melanogaster/D. yakuba* lineage than in *D. melanogaster/D. repleta,* in which element B was the slowest evolving [87]. Thus, in addition to rate variation among lineages, rates of chromosomal evolution may vary across Muller's elements in different groups of *Drosophila,* in good agreement with, for example, the fast evolution of the Muller's element E across the *repleta* species group [40].

**Table 4:**
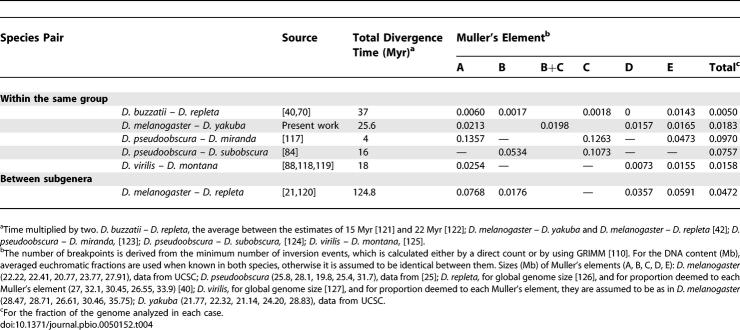
Rates of Chromosomal Evolution (Breakpoints/Mb/Myr) between Different Species Pairs of the Genus *Drosophila*

### Breakpoint Clustering

Breakpoint reuse has been reported at the cytological [54,89–91] and the molecular level [16,25–27,92]. Based on our phylogenetic reconstruction of the chromosomal rearrangements of the species considered here (Table 1), it is clear that some ancestral gene configurations have been disrupted independently more than once during the evolution of the subgenus *Sophophora*. Using sequences from *D. ananassae, D. persimilis,* and D. pseudoobscura as outgroups to the D. melanogaster species subgroup, we found evidence for breakage in 17 out of the 55 (~31%) regions disrupted in the *D. melanogaster/D. yakuba* lineage. We also see evidence for nonrandom breakage in the *D. melanogaster/D. yakuba* complex, i.e., at a relatively short phylogenetic distance. For each of the three pairs of inversions 3L(3)/3L(4), 3R(7)/3R(8), and 3R(10)/3R(11), three, instead of four, breakpoint regions are involved. This recurrent breakage might denote structural instability of particular genomic regions. For example, *CG9579,* one of the genes adjacent to the breakpoints of the inversion X(5), is also linked to a remarkable set of molecular reorganizations associated with the birth of a multigene family of a chimeric gene, *Sdic,* on the D. melanogaster lineage [93]. Additional support for structural instability of inversion breakpoint regions comes from the fact that one breakpoint region of inversion 2LR(4), which occurs on the D. yakuba lineage, uses the same genomic interval that has independently permitted the recent evolution of an unusually high TE density in the D. melanogaster lineage (HDR13 in [94]).

A related issue to breakpoint reuse is the possibility that the same inversion can arise twice. The unique origin of inversions has been challenged (see [37] for discussion), but in the two cases considered to be the most convincing, experimental evidence has not supported a polyphyletic origin of inversions [51,92]. Fourteen breakpoint regions are associated with shared inversions between D. yakuba and D. erecta (Table 1), which indicates that the same gene pairs have been disrupted and reorganized in the same way, suggesting a common origin in the ancestor of D. erecta and D. yakuba. Comparative sequence analysis at the nucleotide level for those 14 junctions failed to find evidence of an independent origin of these inversions in the lineages that lead to the D. yakuba and *D. erecta,* although it must be noted that our power of detection can be compromised by the time elapsed since D. yakuba and D. erecta shared an ancestor.

### Inversion Breakpoints Can Disrupt Large- and Small-Scale Gene Domains

Expression profiling of the genomes of several species has shown that co-expressed genes tend to co-locate in the genome (for review, see [28]). The biological significance of co-expression clustering is still poorly understood, but if these “transcriptional territories” represent functional associations among neighboring genes, natural selection should prevent their disruption. Conservation of clusters across lineages differentiated by the accumulation of multiple chromosomal rearrangements has been interpreted as support for the functional association of clusters of co-expressed genes in mammals [95] and flies [29].

In *D. melanogaster,* the preferential clustering of genes, by the time or place of their expression, has been reported based on both expressed sequence tag (EST) and microarray data [96–99]. In a study of the distribution of sex-biased gene expression [97], 75% of the genes on Release 3.1 of the D. melanogaster genome were assayed. Fifteen gene clusters that are expressed either in testis, in ovary, or in the soma were found. Despite the relatively small number of gene-order interruptions between *D. melanogaster* and *D. yakuba,* one of the clusters identified by Parisi et al. [97], containing the *Try* multigene family, is broken in the lineage of D. yakuba by inversion 2LR(8). At least eight out of ten members of the disrupted gene cluster are highly expressed in the soma. The disruption of this transcriptional territory may be related to the fact that the chromosomal breakage occurred between a member of the cluster, *CG12388 (kappaTry),* which is soma-biased in expression, and *CG12387 (zetaTry),* which is not.

Transcriptional territories have been found to be correlated with the DNA replication program in D. melanogaster [100]. Specifically, 7.5% of the D. melanogaster genome, distributed in 52 well-defined regions, is under-replicated in polytene chromosomes, and 50 of these regions also replicate late during the S period in cultured Kc cells; other regions present a non-delayed replication status in at least one of the two tissues. Sixty percent (30/50) of these late or under-replicating regions are associated with previously defined transcriptional territories; these domains account for 20% of the D. melanogaster genome [98]. Globally, transcriptional territories with a delayed pattern of DNA replication seem to be enriched for genes expressed in the testis and during pupal development, and depleted of genes expressed in the ovary and embryonic development [100]. Are the 55 gene pairs disrupted by inversion breakpoints in the *D. melanogaster/D. yakuba* lineages randomly distributed across the genome with regard to their replication status? We did not find a significant deviation from the random expectation (*G*_adj_= 5.29; *d.f.* = 3; *p* = 0.15); however, we did find that three out of the 53 ancestral gene pairs disrupted in D. yakuba (Xm(8), 2m(19), and 3m(45)) are embedded in regions that are under-replicated in salivary glands and late replicated in Kc cells. These results show that at least some of the regions of the D. melanogaster genome, within which genes have a similar expression profile and/or replication program, are not necessarily conserved between this species and *D. yakuba.* This suggests that either those domains have little adaptive value, supporting the idea of accidental co-expression, or that their adaptive value has evolved recently, relative to the time of the divergence between D. melanogaster and *D. yakuba.*

Some 1,027 pairs of genes in D. melanogaster have overlapping transcripts in opposite strands [101]. Antisense overlap can play an important role in regulating gene expression at the post-transcriptional level [102,103]. Five of these genes pairs are disjunct in *D. yakuba,* as a consequence of an inversion breakpoint. Comparison across lineages (Table 1) indicates that the disruption in D. yakuba represents the derived state. The five inversions that disrupt antisense pairs are all associated with inverted duplications (Table S5). Our model for the origin of inversions (Figure 2) can account for the conservation of sequences of decoupled antisense pairs of genes. At least in two of these cases *(CG9578-CG9579* and *CG31142-CG5289),* the 3′ UTR sequences of the independent gene pairs of D. yakuba are very similar in sequence and in length to their corresponding 3′ UTRs in D. melanogaster. In the other three cases, the D. yakuba 3′ UTR of one of the members of each pair is truncated.

### Conclusion

This work unveils novel aspects of the evolution of the molecular organization of the *Drosophila* genome in particular and of the genomes of insects in general. The use of genome sequence data of D. melanogaster and D. yakuba has proven to be useful in reconstructing the history of genome rearrangements in these species. The lineage that leads to D. yakuba is evolving substantially faster at the chromosomal level than D. melanogaster (28:1); nevertheless, the mechanism that underlies the generation of many inversions (~59%) in both lineages is the same, and it seems to be initiated by the presence of staggered breaks, which in turn enables the generation of duplications in inverted orientation of sequences at co-occurrent breakpoint regions. These duplications diverge mainly by both nucleotide substitutions and small deletions [104,105], and can contribute, as do segmental duplications in mammals, to the diversification of gene function [106]. A model of inversion generation based on staggered breaks, either isochromatid or chromatid, contrasts with a model of ectopic recombination between repetitive sequences [46,75,76]. Our data also give clear evidence, at the molecular level, of the reuse of the same breakpoint region and that expression domains in D. melanogaster may be disrupted in other species, bringing into question their potential adaptive significance.

The availability of complete sequences from 12 *Drosophila* species now offers the opportunity to extend the analysis of chromosome evolution at a molecular level. Several fundamental questions remain: whether or not mechanisms of inversion formation are general across taxa; and whether there are functional constraints on chromosomal evolution, and, if so, at what level do these operate.

## Materials and Methods

### Flies.

The following species and strains were used: D. melanogaster (OR-R from the Department of Genetics, University of Cambridge, and Zimbabwe 2 from D. L. Hartl's laboratory); D. simulans (Sim-1 from Chapel Hill, North Carolina); and D. yakuba (Tai18E2 from the Tucson Stock Center). In the case of Zimbabwe 2 and Tai18E2, we checked whether they were homokaryotypic by visually examining salivary gland polytene chromosome preparations stained with orcein. In the case of Zimbabwe 2, we detected two paracentric inversions in a sample of 20 autosomal genomes and 16 X chromosome genomes. No gross chromosomal polymorphisms were detected in a sample of 20 autosomal genomes and 16 X chromosome genomes of Tai18E2.

### In situ hybridization of molecular probes onto polytene chromosomes.

Five BACs and 11 genomic clones were used as molecular probes. The BAC clones (BACR07M14, BACR45A07, BACR16N15, BACR42I20, and BACR08K01) were obtained from the Children's Hospital Oakland Research Institute. Genomic clones were PCR amplified using the primers described in Table S7. The genomic DNA used for the PCR amplifications was from the sequenced strain of *D. melanogaster: y; cn bw sp* [107]. The genomic fragments generated correspond to the protein-coding genes *CG2708 (Tom34), CG7918, CG31176, CG34034, CG5289,* and *CG6576 (Glec);* the putatively transcribed genes *HDC14860, HDC14861, HDC14862,* and *HDC12400* [60]; and the sequence of *pdf800,* which is said to be related to the mammalian proto-oncogene c*-fos*. Cloning of PCR products and preparation of DNA from recombinant clones was performed using conventional methods. In the case of BAC clones, we used the methods described at http://bacpac.chori.org/bacpacmini.htm. In situ hybridization of probes to polytene chromosomes was done as in [108]. Detection of the hybridization signals was done by phase contrast with a Zeiss Axioskop 2 (Carl Zeiss, http://www.zeiss.com). Chromosomal localization was determined using the photographic polytene chromosome maps of D. melanogaster [109]. All the probes yielded one or two hybridization signals with the exception of those for *HDC14860* and *HDC14861,* which failed to generate a detectable hybridization signal in D. yakuba under the experimental conditions used.

### Assembly of D. yakuba supercontigs into chromosomal sequences.

The sequencing and assembly of the D. yakuba genome will be described elsewhere (D. J. Begun, A. K. Holloway, K. Stevens, L. W. Hillier, Y.-P. Poh, M. W. Hahn, P. M. Nista, C. D. Jones, A. D. Kern, C. Dewey, L. Pachter, E. Myers, and C. H. Langley, unpublished data). To create chromosomal assignments and ordering of “supercontigs” (gapped scaffolds of ungapped contigs as defined by mate pairs) along the chromosomes for the D. yakuba genome assembly, contigs from the D. yakuba assembly that uniquely aligned with the D. melanogaster genome were identified and then ordered by their positions along the assigned D. melanogaster chromosomes. This process resulted in some D. yakuba supercontigs with contigs that aligned to different regions of a D. melanogaster chromosome. To assemble supercontigs into chromosome arms in *D. yakuba,* reversals of the tiling path of mapped contigs were introduced to “rejoin” those supercontigs that had been split by the alignments to *D. melanogaster.* The overall goal was to minimize the total number of reversals required to rejoin all D. yakuba supercontigs previously assigned to disjoint chromosomal regions based on D. melanogaster alignments. We note that reversals were introduced only between contigs (not within contigs) and the process was not gene based.

### Gene-order reconstruction in *D. yakuba.*

The complete set of transcripts of the D. melanogaster Release 4.1 annotation was downloaded from UCSC Genome Browser (http://genome.ucsc.edu/). This set represents 13,449 annotated genes. Each D. melanogaster transcript was used as a query against the assembly of the D. yakuba genome release 2.0 (WUSTL November 2005, the droYak2 assembly) using BLASTN 2.2.2 with default settings and then filtered for the top hit for each transcript with a cutoff E-value of 10^−30^; the nonfiltered output can be found as Table S8. This approach localized 12,690 genes on the genome sequence of D. yakuba with a best hit on the same chromosome arm (with exceptions made for genes inside the pericentric inversion on chromosome 2); 320 genes had no BLASTN hit higher than 10^−30^, and 429 genes hit unmapped scaffolds or gave multiple hits with equal E-value in more than one chromosome arm. Genes unambiguously localized were sorted into chromosome order (centromere to telomere) for the six Muller's elements of D. yakuba. The gene order in D. yakuba was compared with that of *D. melanogaster,* and gene-order interruptions between the two species were inferred; the two genes flanking each gene-order interruption were taken as the limits of different syntenic blocks. This method will not reliably detect very small rearrangements, although we know that these occur (e.g., Figure S7; see also [72]). For calculating the minimum number of inversions necessary to transform the gene order of D. melanogaster into that of *D. yakuba,* we used GRIMM [110]. Estimates on the size of syntenic blocks and regions between them in D. yakuba were obtained by taking into account the coordinates of the BLASTN hits of the outermost markers of each syntenic block. In the case of transposition events, we examined the nonfiltered output for genes whose BLAST hits were surrounded by different pairs of flanking genes in D. melanogaster and *D. yakuba,* especially those with unambiguous hits in different Muller's elements.

One complicating factor in our analysis is that BLASTN of a region including 3R:3862326–3867817 was highly similar to two different regions of the D. yakuba assembly: one on Contig690 (currently assembled into chromosome arm 3R), and one, with a slightly lower match, on Contig706 (currently assigned to the “random” bin of chromosome arm 3R because it seemed to overlap Contig690). Contig690 has a sequence coverage of 5.8–8.3×, Contig706 of 3–4.7×. The overall coverage of the genome is 9.4×, but the supercontigs of chromosome arms 2R and 3R have approximately 12× coverage. Were this region to be truly duplicated in the genome of *D. yakuba,* we would expect the sum of the coverage of Contigs 690 and 706 to be at the very least 18×, rather than (at most) 13×. In situ hybridization to polytene chromosomes of probes from this region shows only a single site, that expected on chromosome arm 3R. Residual heterozygosity for other regions of the D. yakuba sequence has been experimentally verified (J. Comeron and C. Langley, personal communication), and we interpret these two hits as being the consequence of heterozygosity in the genome.

### Experimental verification of the molecular organization at breakpoint regions in D. yakuba.

To confirm the predicted gene-order interruptions between D. melanogaster and *D. yakuba,* we cloned and sequence verified the transition between adjacent syntenic blocks of 27 (49%) of the breakpoint regions in *D. yakuba,* namely Xy(9), Xy(10), 2y(19–24), 2y(26–28), 3y(35–43), 3y(46–51), and 3y(53) (Table S1). We extracted genomic DNA from the sequenced strain Tai18E2 by conventional methods. We designed primers to amplify the sequence that spans the transition between syntenic blocks. In a few cases, either because of the size of the region between the neighboring reference genes or because of technical difficulties, we amplified sets of overlapping segments that ensured coverage of the transition between adjacent syntenic blocks. PCR products were cloned into a pCR2.1 Topo Vector (Invitrogen, http://www.invitrogen.com). Sequencing reactions of the two ends of each clone were done, and the reads were aligned by BLAST against the D. melanogaster genome. Primers used are listed in Table S7.

### Sequence analysis of breakpoint regions in D. yakuba and *D. melanogaster.*

Because not all the genes of D. melanogaster were mapped to the D. yakuba assembly, and because there may have been transpositions of regions during the evolution of these genomes, we extracted the sequences of the 55 genomic discontinuities of *D. yakuba,* relative to *D. melanogaster,* and aligned these by BLASTN against the D. melanogaster genome. This refined the limits of the syntenic blocks and allowed their ends to be precisely mapped. To identify duplicates at co-occurrent breakpoint regions, we used PipMaker [111], and BLAST2 [112] with their default parameters. Sequences from all local alignments spanning more than 40 bp from PipMaker were used as queries in a BLASTN analysis against the D. melanogaster genome, thereby verifying their identities and genomic locations. We did the same with the BLAST2 output for those sequences with hits whose E-value were lower than 10^−8^ and were at least 40-bp long. Both approaches provided essentially the same results. Nucleotide identities between particular duplicates and their reference sequences were derived from the BLAST2 analysis. For genes that are adjacent to breakpoints and/or are affected by them, we did an additional BLAST2 analysis, using as queries the D. melanogaster sequences of their transcripts. Sequences that are now found as inverted duplications at co-occurrent breakpoint regions may not necessarily have been in this orientation immediately after the occurrence of the inversion, because subsequent events may have taken place. For this reason, we reconstructed the most parsimonious history of each inversion in an attempt to establish the sequence immediately after each had occurred. We analyzed the presence of TE sequences using the RepeatMasker track from UCSC (RepBase libraries: RepBase Update 9.11 and RM database version 20050112) and subsequently by BLAST2 analysis using a collection of TE sequences that includes those in different *Drosophila* species other than *D. melanogaster.* All the significant hits found by our BLAST2 analysis correspond to footprints of TEs of D. melanogaster previously detected with RepeatMasker. For duplications that spanned noncoding regions, we did a BLASTN analysis against the D. yakuba genome, in order to determine that they did not include repetitive sequences. When necessary, we proceeded in an identical manner with breakpoint regions of *D. melanogaster, D. simulans,* and D. erecta.

### Phylogenetic status of the gene configurations at breakpoint regions of D. melanogaster and *D. yakuba.*

In order to determine whether the gene configuration in the breakpoint regions in D. melanogaster or in D. yakuba is ancestral or derived, i.e., the result of a chromosomal rearrangement, we took D. melanogaster as a reference, and we determined whether or not the reference genes within a particular breakpoint region were adjacent in a set of species selected on the basis of their phylogenetic relationships with D. melanogaster and D. yakuba. Specifically, we used: D. melanogaster (Release 4.1; FlyBase); D. simulans (release 1.0 Apr. 2005; UCSC); D. yakuba (droYak2 Nov. 2005); D. erecta (droEre1 Aug. 2005; UCSC); D. ananassae (droAna2 Aug. 2005; UCSC); D. persimilis (droPer1 Oct. 2005 UCSC); and D. pseudoobscura (Release 1.0; S. W. Schaeffer, personal communication). We used PipMaker to analyze the breakpoint regions apparently shared between D. yakuba and *D. erecta.* If these breakpoint regions were of *independent* origin, then we would expect to see discontinuities and indels between them. In fact, in all cases, the evidence suggests that these “shared” breakpoints were the consequence of a single ancestral event.

## Supporting Information

Figure S1 Phylogenetic Relationships in the Genus *Drosophila* and Its Subgenera: *Drosophila* and *Sophophora*
The phylogenetic relationships among the species used in the present study are shown in detail. All belong to the subgenus *Sophophora*. The *melanogaster* species subgroup comprises nine species, which have been commonly clustered into two complexes by the criteria of gene sequences, polytene chromosome banding pattern, and the structures of the male genitalia [54,113–115]. One of the complexes includes *D. melanogaster* and the trio *D. mauritiana, D. sechellia,* and *D. simulans,* and the second *D. erecta, D. orena, D. santomea, D. teissieri,* and D. yakuba. All the divergences times are according to [42].
(12 KB PDF)

Figure S2 The Isochromatid Model with Staggered Single-Strand Breaks in the Case of the Inversion 3R(7) (A) Relative to the gene order of *D. simulans,* the region from *CG15179* to *CG17603* is inverted, due to a prior event (dotted line).
(B and C) Inversion 3R(7) originates from two pairs of staggered single-strand breaks (short horizontal solid lines), proximally on either side of *CG31286,* and distally on either side of *CG34034.* The resulting 5′-overhangs are filled in (grey dashed arrow) and followed by a nonhomologous end joining.
(D) As a consequence, both *CG34034* and *CG31286* were duplicated at both breakpoints.
(E) Subsequently, both *CG34034* and *CG31286* tandemly duplicated, before other mutations affected both copies of *CG31286,* one copy of *CG34034,* and the *HDC14862*(5′) sequence.
These events illustrate the complexity of some inversion breakpoint regions as a consequence of events that occur subsequent to the original inversion. Color code as in Figure 1. For the sake of simplicity, two putatively expressed genes *(HDC12142* and *HDC12143)* and insertions of repetitive sequences have not been included.
(28 KB PDF)

Figure S3 A Chromatid Model with Staggered Double-Strand Breaks Can Also Give Rise to an Inversion Accompanied by Inverted Duplications of Sequences Included in the Breakpoint Regions The mechanism is illustrated by the inversion 3R(8), which is fixed in the lineage to D. melanogaster.
(A) Sister chromatids in meiotic prophase showing the gene order and orientation assumed to be ancestral, which is currently best represented by D. simulans (Figure 1).
(B) Two pairs of staggered double-strand breaks *(a-b* and *c-d)* are indicated.
(C) Nonhomologous end joining results in two chromatids: one carrying an inversion flanked by inverted duplications of the sequences between the paired double-strand breaks, and a second with reciprocal deletions.
Landmarks: A, *CG2708;* B, *HDC14862* (3′); C, *pfd800;* D, *HDC12400;* E, *HDC14861;* F, *HDC14861;* G, *CG31176;* H, *CG7918;* I, *HDC14862* (5′); J, *CG34034;* and K, *CG5849*. Color code as in Figure 1. Black circle indicates the centromere.
(35 KB PDF)

Figure S4 Large-Scale Comparison of the Muller's Elements A, D, and E between D. melanogaster and D. yakuba
(A), Muller's element A (chromosome X); (B) Muller's elements D and E (chromosome 3). The outermost protein-coding genes of consecutive syntenic blocks are indicated. Following [116], syntenic blocks (defined as regions in which the relative gene order is globally conserved between D. melanogaster and D. yakuba) are numbered taking D. melanogaster as a reference and in an increasing order from the telomere of chromosome X (number 1) to the telomere of the right arm of chromosome 3 (number 58); an arrowhead indicates the orientation of the segments. Lines between chromosomes match homologous syntenic blocks between species. Solid triangles correspond to genes that were duplicated during the generation of inversions in the lineage that leads to D. yakuba following a model of staggered strand breaks (Figures 2, S2, and S3). Those genes are *CG14187,* which was generated by the inversion X(1) in (A), and *CG34034,* which was generated by the inversion 3R(7) in (B). Open triangle denotes gene *CG9925,* whose relocation can be explained by a conservative transposition event or, alternatively, by two paracentric inversions that overlap by one gene, *CG9925*. The fact that *CG9925* is flanked both in D. melanogaster and D. yakuba by genes that, in their turn, are the outermost markers of different syntenic blocks strongly supports the second explanation.
(34 KB PDF)

Figure S5 Different Evolutionary Scenarios That Can Lead to the Presence of Inversion-Mediated Duplications at Only One of the Two Co-occurrent Breakpoint Regions The inversion X(1) is used as an example. D. melanogaster (top gene configuration) and D. yakuba (bottom gene configuration).
(A) Scenario involving four staggered breakpoints (arrows). In this case, the duplication of *CG14817* and *HDC18578* is coupled with the generation of the inversion. Subsequently, one of the copies of *HDC18578* degenerates by accumulating nucleotide substitutions and indels so that it is no longer recognizable.
(B) Scenario involving staggered breakpoints at one genomic region and a single-strand break at the other. In this case, only *CG14817* becomes duplicated as a result of the inversion.
The outcome of both scenarios is identical. Coding sequences that have undergone an inversion-mediated duplication in the lineage that leads to *D. yakuba, CG14817* (in green) and *HDC18578* (in pink) are indicated by a gradient.
C, centromere; T, telomere.
(25 KB PDF)

Figure S6 Inversions Required to Transform the Gene Arrangement of Chromosome 2 between D. melanogaster and D. yakuba
The diagram shows 11 inversions, one pericentric and ten paracentric. Other scenarios obtained with GRIMM involve the same number of reversals of gene order [110]. Duplications at breakpoint regions, disruption of multigene families and antisense overlapping, and gene organization in outgroup species are the criteria used to infer the polarization (Table 1). Using this information, the inversions 2L(3), 2R(11), and 2LR(5) occurred first because all are shared between D. yakuba and D. erecta. Note that the order of these inversions is arbitrary. The other inversions took place after the split of the lineage that lead to D. yakuba and D. erecta. The numbering of the syntenic blocks follows that of Figure 3; the blocks of D. yakuba appear with a minus sign if inverted in relation to D. melanogaster.
(52 KB PDF)

Figure S7 Dot Plot for the Genomic Sequence of the Syntenic Block 42 between D. melanogaster and D. yakuba
A few cases of departures from perfect collinearity are observed denoting small rearrangements. The one on the upper right corner is an inversion involving at least four genes: *CG12284, CG5895, CG13076,* and *CG5830*. The dot plot was generated with PipMaker [111]. The genome sequences spanning from the gene *CG6749* to the gene *CG32147,* both in D. melanogaster and in *D. yakuba,* were extracted from UCSC. The sizes of block 42 in each species are indicated on the corresponding axes.
(311 KB PDF)

Table S1 Co-occurrent Breakpoint Regions of the Inversions between D. melanogaster and D. yakuba
(200 KB RTF)

Table S2 Size of Syntenic Blocks between D. melanogaster and D. yakuba with the Number of Genes That Have, or Have Not, Been Mapped between Them (132 KB RTF)

Table S3 Conservative Transposition Events Detected between D. melanogaster and D. yakuba
(17 KB RTF)

Table S4 Duplicative Transposition Events Detected between D. melanogaster and D. yakuba
(52 KB RTF)

Table S5 List of Duplications of Nonrepetitive DNA Sequences Present at Breakpoint Regions of Inversions between D. melanogaster and D. yakuba
(47 KB XLS)

Table S6 Repeat Composition Characterization by RepeatMasker in Co-occurrent Breakpoint Regions of D. melanogaster and D. yakuba
(211 KB RTF)

Table S7 Primers Used in This Work for Cloning and/or Sequencing (135 KB RTF)

Table S8 Nonfiltered Output of the BLASTN of the D. melanogaster Transcripts against the D. yakuba Assembly (17.5 MB XLS)

### Accession Numbers

The GenBank (http://www.ncbi.nlm.nih.gov/Genbank) accession number for the D. melanogaster DNA sequence *pfd800* discussed in this paper is Z16407. The accession numbers for the sequences generated in this paper are EF569486–EF569554.

## Abbreviations

BAC: bacterial artificial chromosome
bp: base pair
kb: kilobase
Mb: megabase
Myr: million years
TE: transposable element
UCSC: the University of California, Santa Cruz
