# Supplementary material for: Principles of Genome Evolution in the Drosophila melanogaster Species Group
Source: PLoS Biol. 2007 Jun 5;5(6):e152. doi: 10.1371/journal.pbio.0050152 (PMC1885836; doi:10.1371/journal.pbio.0050152)
Supplement: Supplementary file 1 [file pbio.0050152.sg001.pdf]

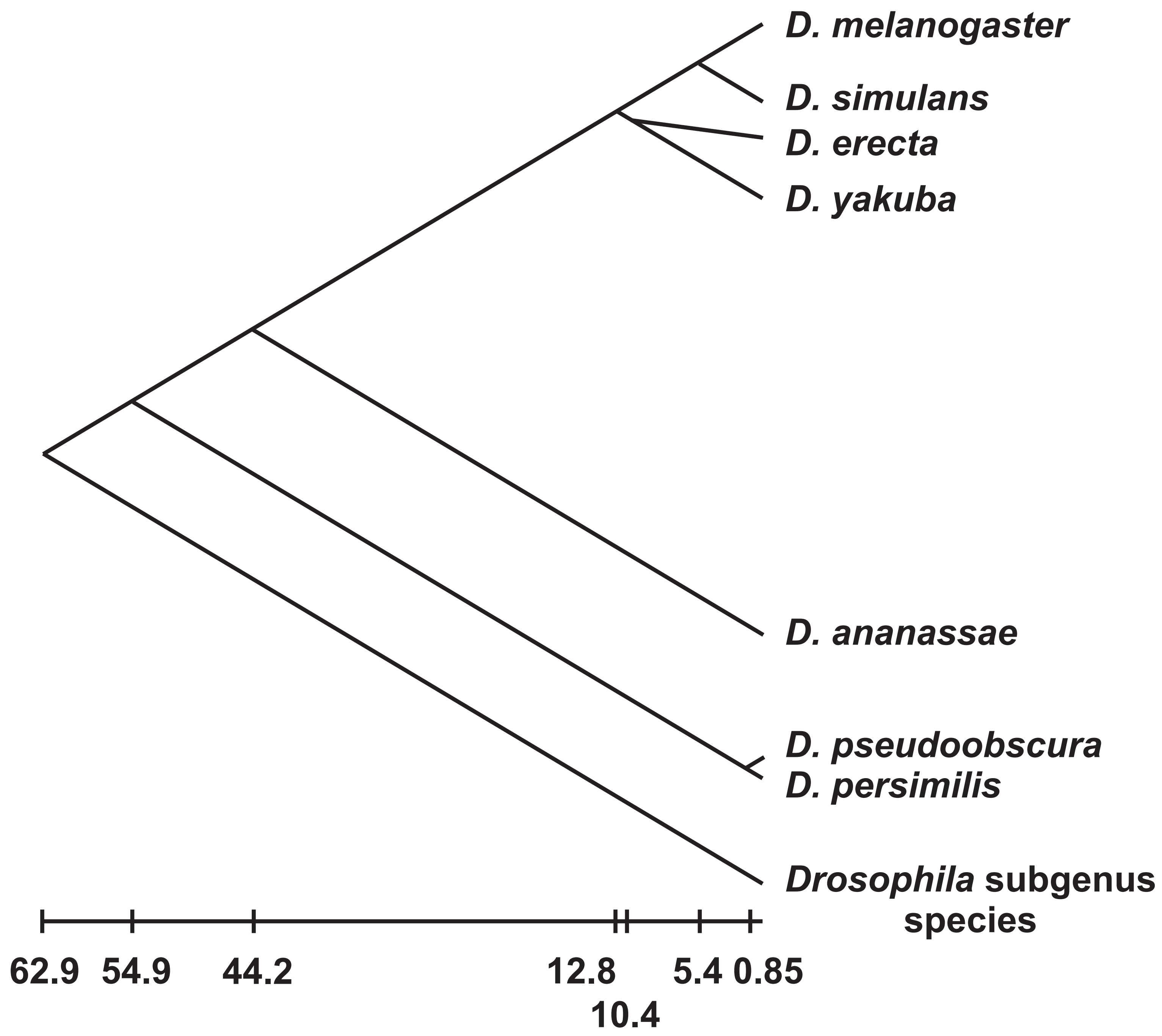
D. melanogaster
D. simulans
D. erecta
D. yakuba
D. ananassae
D. pseudoobscura
D. persimilis
Drosophila subgenus species
62.9
54.9
44.2
12.8
10.4
5.4
0.85
