# Supplementary material for: Principles of Genome Evolution in the Drosophila melanogaster Species Group
Source: PLoS Biol. 2007 Jun 5;5(6):e152. doi: 10.1371/journal.pbio.0050152 (PMC1885836; doi:10.1371/journal.pbio.0050152)
Supplement: Supplementary file 2 [file pbio.0050152.sg002.pdf]

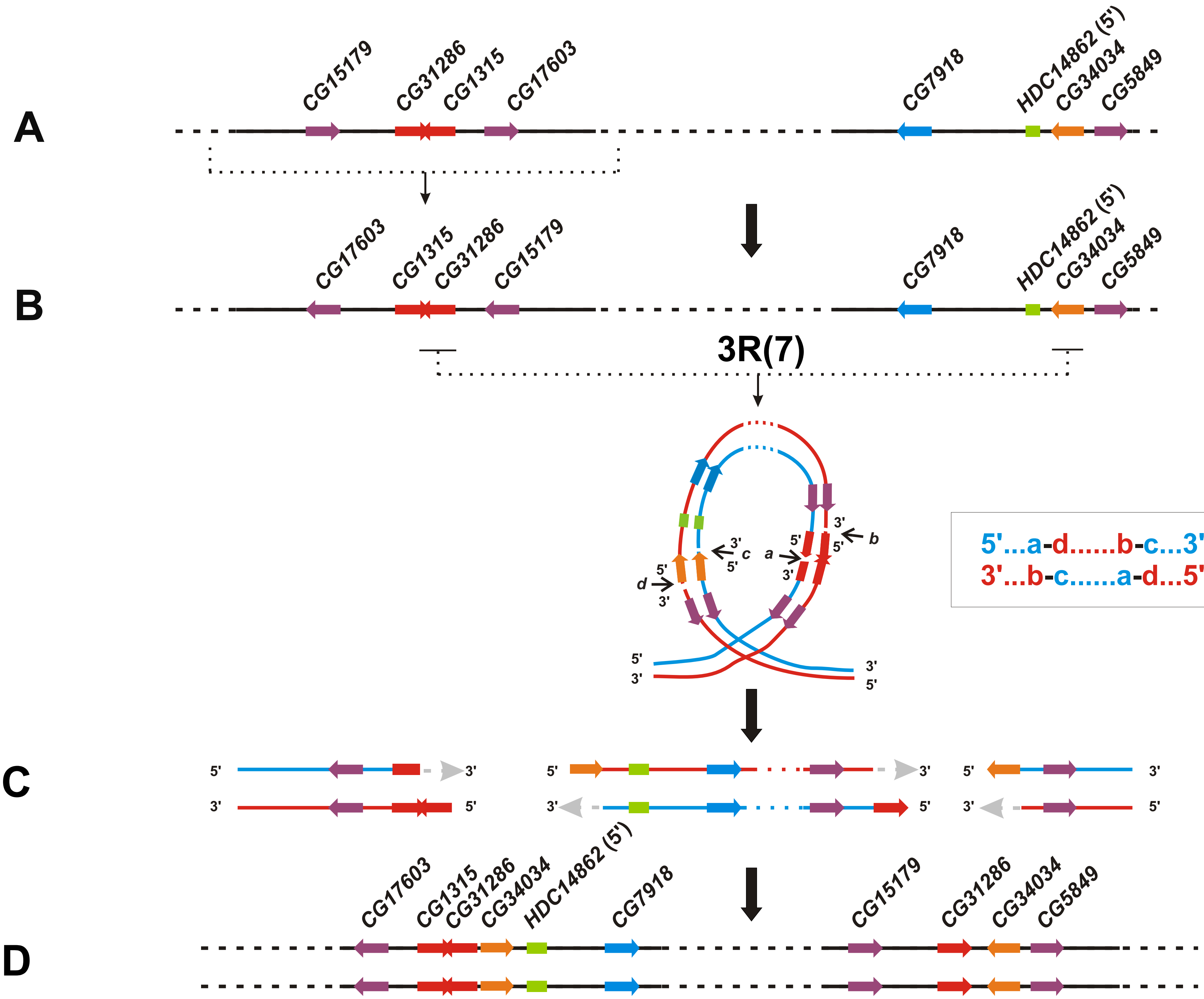
A
CG15179
CG31286
CG1315
CG17603
CG7918
HDC14862 (5')
CG34034
CG5849
B
CG17603
CG1315
CG31286
CG15179
CG7918
HDC14862 (5')
CG34034
CG5849
3R(7)
5'...a-d......b-c...3'
3'...b-c......a-d...5'
C
D
CG17603
CG1315
CG31286
CG34034
HDC14862 (5')
CG7918
CG15179
CG31286
CG34034
CG5849

D

E

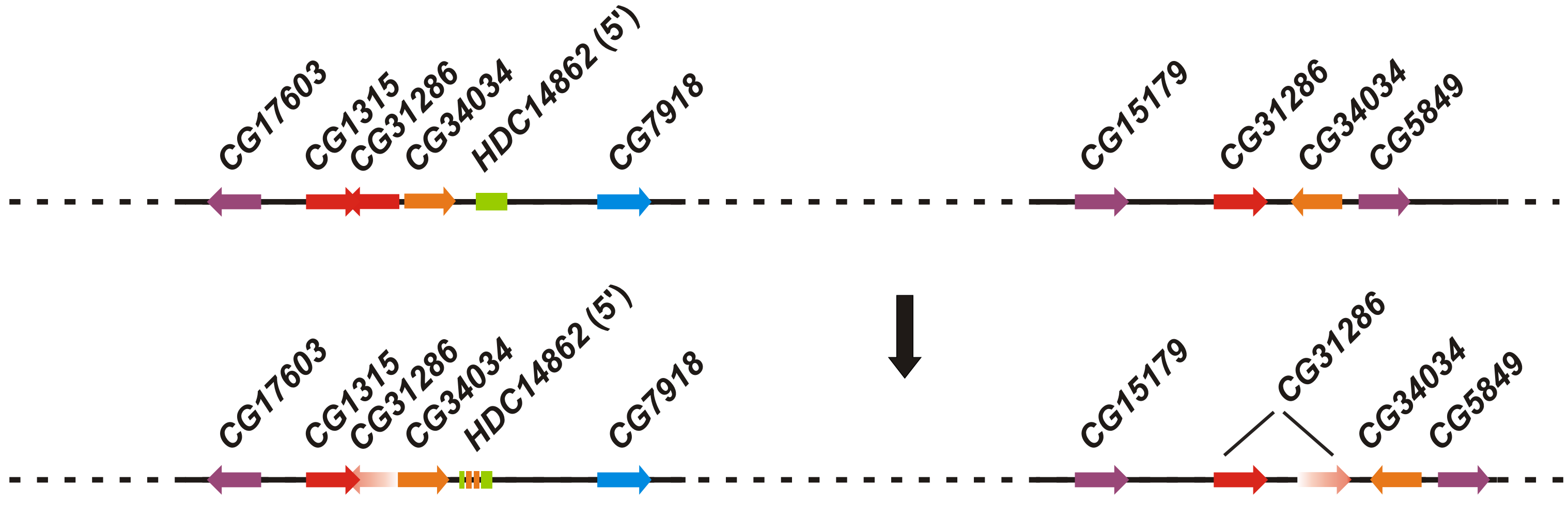

CG17603
CG1315
CG31286
CG34034
HDC14862 (5')
CG7918
CG15179
CG31286
CG34034
CG5849
CG17603
CG1315
CG31286
CG34034
HDC14862 (5')
CG7918
CG15179
CG31286
CG34034
CG5849
