# Supplementary material for: Principles of Genome Evolution in the Drosophila melanogaster Species Group
Source: PLoS Biol. 2007 Jun 5;5(6):e152. doi: 10.1371/journal.pbio.0050152 (PMC1885836; doi:10.1371/journal.pbio.0050152)
Supplement: Supplementary file 3 [file pbio.0050152.sg003.pdf]

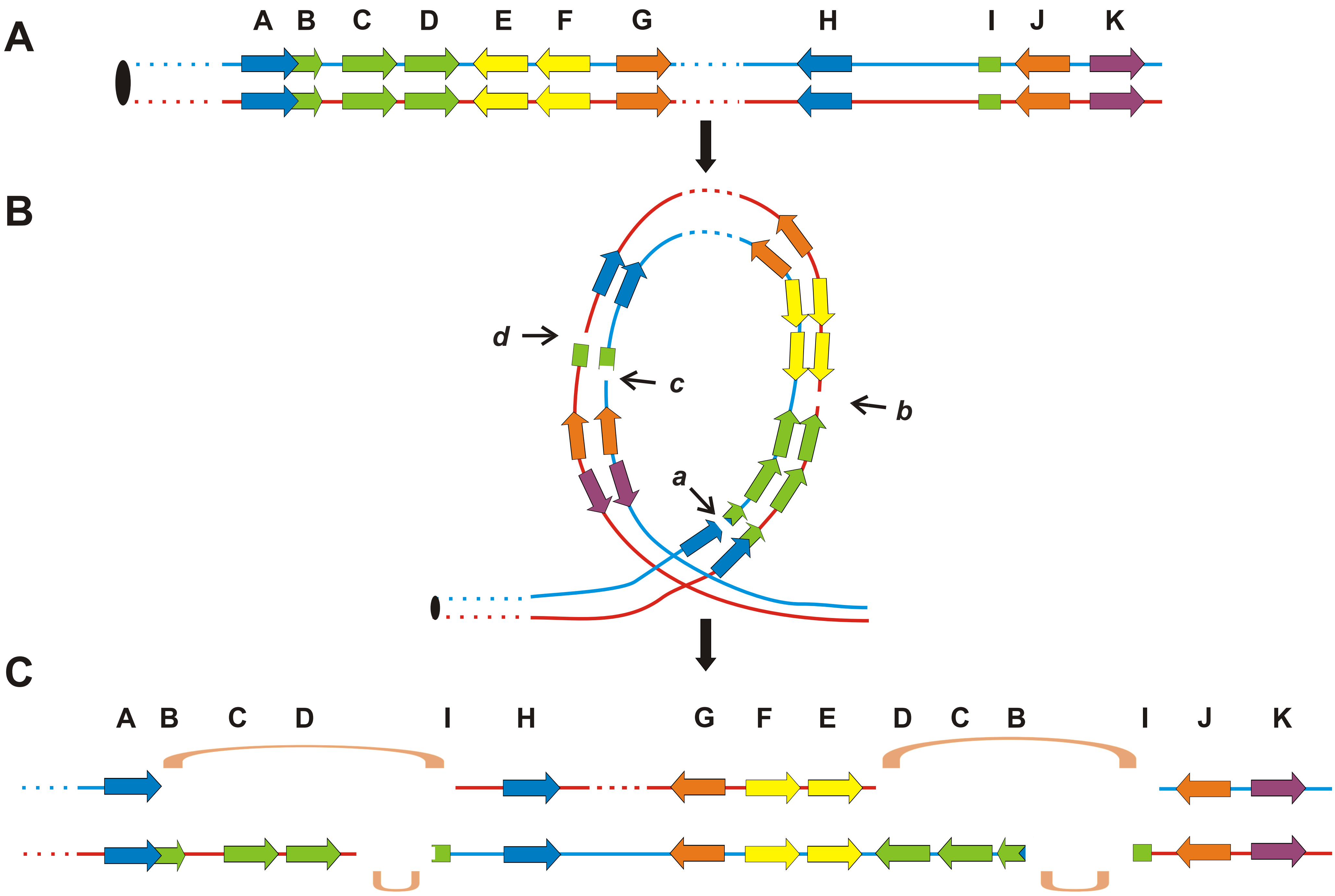
A
A B C D E F G H I J K
B
d
c
b
a
C
A B C D I H G F E D C B I J K
