# Supplementary material for: Principles of Genome Evolution in the Drosophila melanogaster Species Group
Source: PLoS Biol. 2007 Jun 5;5(6):e152. doi: 10.1371/journal.pbio.0050152 (PMC1885836; doi:10.1371/journal.pbio.0050152)
Supplement: Supplementary file 4 [file pbio.0050152.sg004.pdf]

A

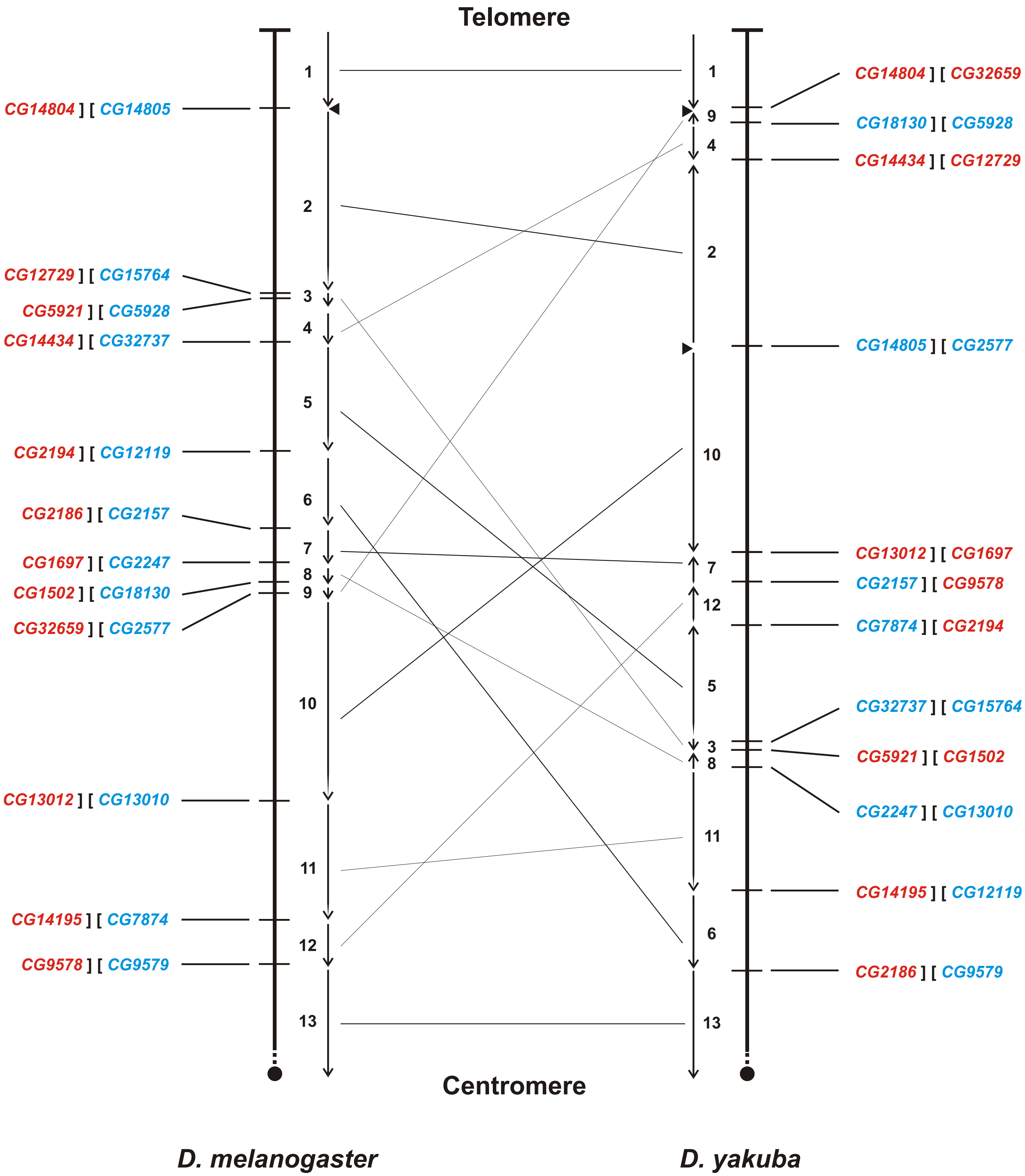

Telomere
Centromere
CG14804] [CG14805
CG12729] [CG15764
CG5921] [CG5928
CG14434] [CG32737
CG2194] [CG12119
CG2186] [CG2157
CG1697] [CG2247
CG1502] [CG18130
CG32659] [CG2577
CG13012] [CG13010
CG14195] [CG7874
CG9578] [CG9579
1
2
3
4
5
6
7
8
9
10
11
12
13
1
9
4
2
10
7
12
5
3
8
11
6
13
CG14804] [CG32659
CG18130] [CG5928
CG14434] [CG12729
CG14805] [CG2577
CG13012] [CG1697
CG2157] [CG9578
CG7874] [CG2194
CG32737] [CG15764
CG5921] [CG1502
CG2247] [CG13010
CG14195] [CG12119
CG2186] [CG9579
D. melanogaster
D. yakuba

X

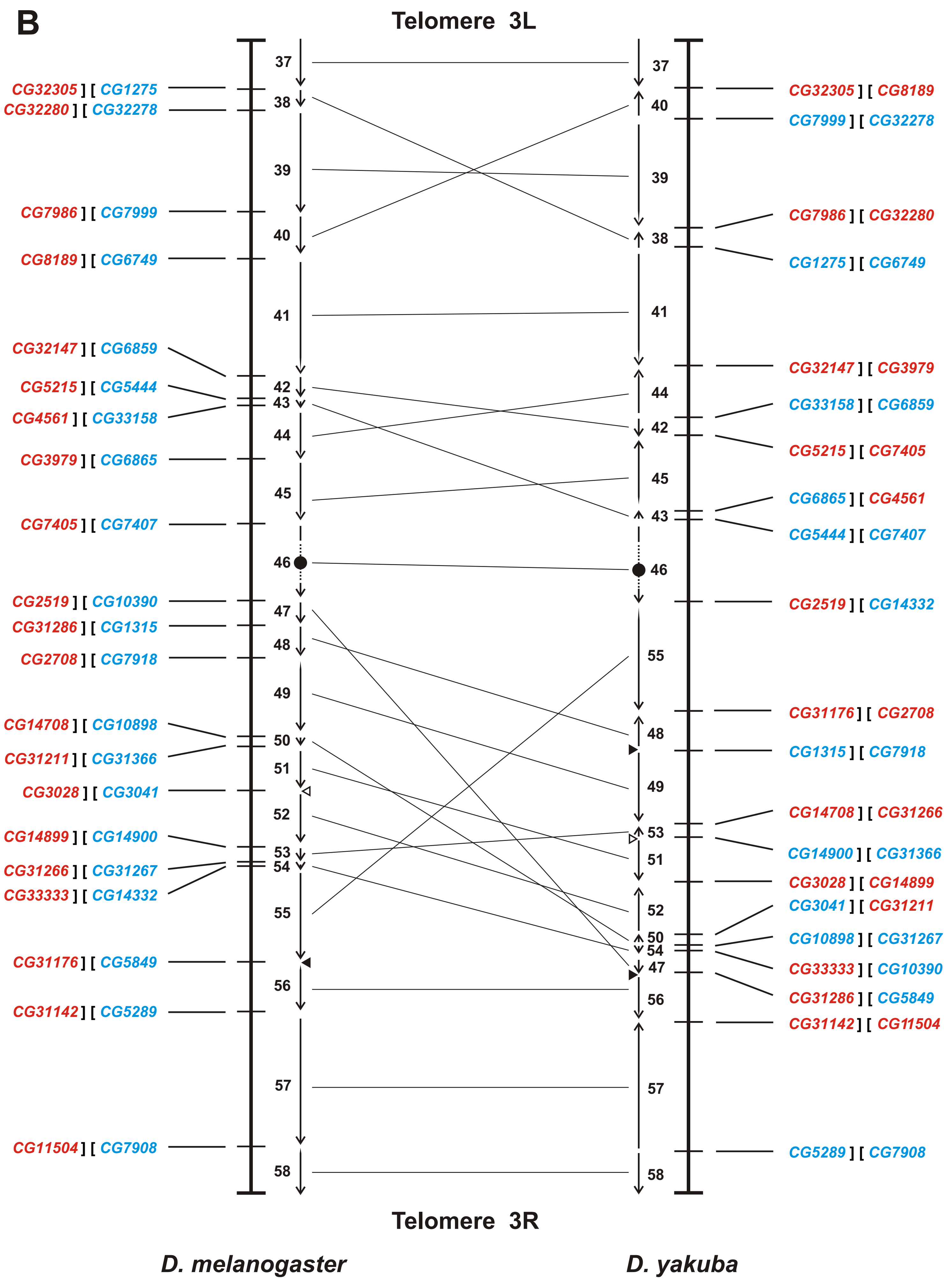
B
Telomere 3L
Telomere 3R
D. melanogaster
D. yakuba
CG32305] [CG1275
CG32280] [CG32278
CG7986] [CG7999
CG8189] [CG6749
CG32147] [CG6859
CG5215] [CG5444
CG4561] [CG33158
CG3979] [CG6865
CG7405] [CG7407
CG2519] [CG10390
CG31286] [CG1315
CG2708] [CG7918
CG14708] [CG10898
CG31211] [CG31366
CG3028] [CG3041
CG14899] [CG14900
CG31266] [CG31267
CG33333] [CG14332
CG31176] [CG5849
CG31142] [CG5289
CG11504] [CG7908
CG32305] [CG8189
CG7999] [CG32278
CG7986] [CG32280
CG1275] [CG6749
CG32147] [CG3979
CG33158] [CG6859
CG5215] [CG7405
CG6865] [CG4561
CG5444] [CG7407
CG2519] [CG14332
CG31176] [CG2708
CG1315] [CG7918
CG14708] [CG31266
CG14900] [CG31366
CG3028] [CG14899
CG3041] [CG31211
CG10898] [CG31267
CG33333] [CG10390
CG31286] [CG5849
CG31142] [CG11504
CG5289] [CG7908
37
38
39
40
41
42
43
44
45
46
47
48
49
50
51
52
53
54
55
56
57
58
