# Supplementary material for: Principles of Genome Evolution in the Drosophila melanogaster Species Group
Source: PLoS Biol. 2007 Jun 5;5(6):e152. doi: 10.1371/journal.pbio.0050152 (PMC1885836; doi:10.1371/journal.pbio.0050152)
Supplement: Supplementary file 5 [file pbio.0050152.sg005.pdf]

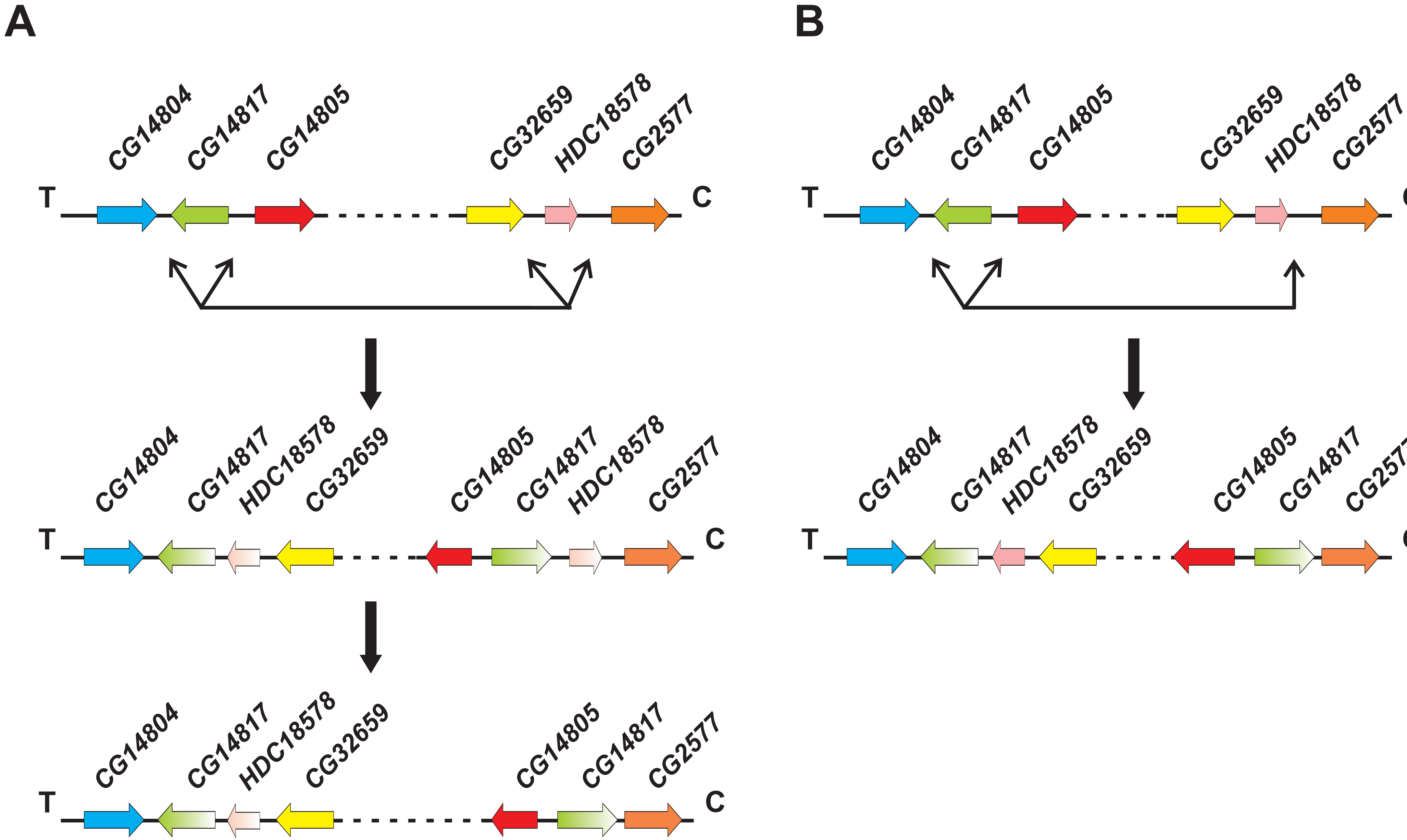
A
CG14804
CG14817
CG14805
CG32659
HDC18578
CG2577
T
C
CG14804
CG14817
HDC18578
CG32659
CG14805
CG14817
HDC18578
CG2577
T
C
CG14804
CG14817
HDC18578
CG32659
CG14805
CG14817
CG2577
T
C
B
CG14804
CG14817
CG14805
CG32659
HDC18578
CG2577
T
C
CG14804
CG14817
HDC18578
CG32659
CG14805
CG14817
CG2577
T
C
