# Supplementary material for: Principles of Genome Evolution in the Drosophila melanogaster Species Group
Source: PLoS Biol. 2007 Jun 5;5(6):e152. doi: 10.1371/journal.pbio.0050152 (PMC1885836; doi:10.1371/journal.pbio.0050152)
Supplement: Supplementary file 6 [file pbio.0050152.sg006.pdf]

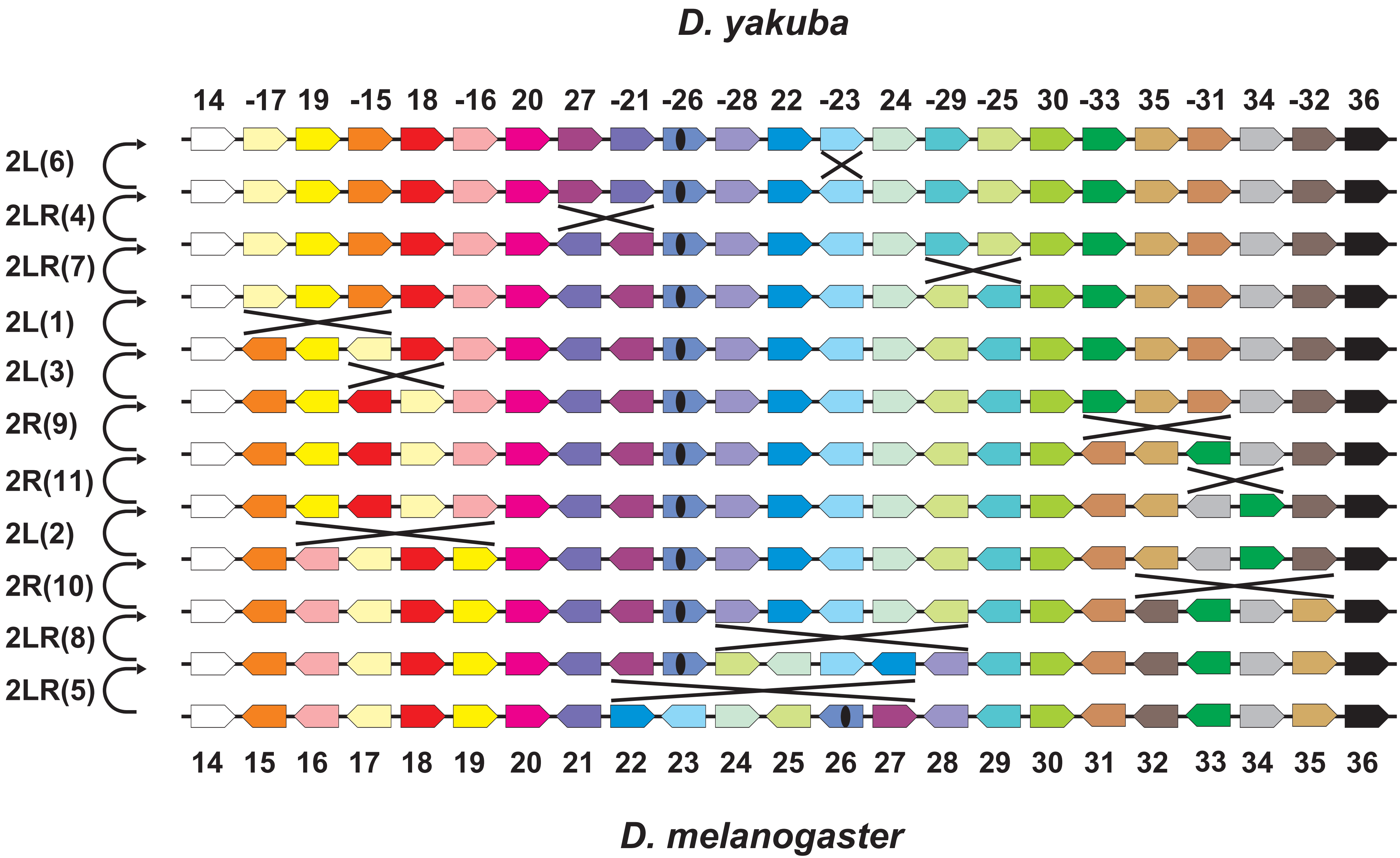
D. yakuba
14 -17 19 -15 18 -16 20 27 -21 -26 -28 22 -23 24 -29 -25 30 -33 35 -31 34 -32 36
2L(6)
2LR(4)
2LR(7)
2L(1)
2L(3)
2R(9)
2R(11)
2L(2)
2R(10)
2LR(8)
2LR(5)
14 15 16 17 18 19 20 21 22 23 24 25 26 27 28 29 30 31 32 33 34 35 36
D. melanogaster
