# Supplementary material for: Principles of Genome Evolution in the Drosophila melanogaster Species Group
Source: PLoS Biol. 2007 Jun 5;5(6):e152. doi: 10.1371/journal.pbio.0050152 (PMC1885836; doi:10.1371/journal.pbio.0050152)
Supplement: Supplementary file 8 [file pbio.0050152.st001.rtf]

TABLE S1.  Co-occurrent breakpoint regions of the inversions between D. melanogaster and D. yakuba.	
	Breakpoints  Regions	
	D. melanogaster	D. yakuba	
Inversion		 Code	Reference Genes		 Code	Reference Genes	
X(1)			Xm(1)	CG14804 - CG14805		Xy(1)	CG14804 - CG32659	
			Xm(9)	CG32659 - CG2577		Xy(4)	CG14805 - CG2577	
								
X(2)			Xm(2)	CG12729 - CG15764		Xy(3)	CG14434 - CG12729	
			Xm(4)	CG14434 - CG32737		Xy(8)	CG32737 - CG15764	
								
X(3)			Xm(3)	CG5921 - CG5928		Xy(2)	CG18130 - CG5928	
			Xm(8)	CG1502 - CG18130		Xy(9) ^1^	CG5921 - CG1502	
								
X(4)			Xm(5)	CG2194 - CG12119		Xy(7)	CG7874 - CG2194	
			Xm(11)	CG14195 - CG7874		Xy(11)	CG14195 - CG12119	
								
X(5)			Xm(6)	CG2186 - CG2157		Xy(6)	CG2157 - CG9578	
			Xm(12)	CG9578 - CG9579		Xy(12)	CG2186 - CG9579	
								
X(6)			Xm(7)	CG1697 - CG2247		Xy(5)	CG13012 - CG1697	
			Xm(10)	CG13012 - CG13010		Xy(10) ^1^	CG2247 - CG13010	
								
2L(1)			2m(13)	CG3036 - CG2837		2y(13)	CG3036 - CG7233	
			2m(16)	CG7233 - CG7231		2y(16)	CG2837 - CG7231	
								
2L(2)			2m(14)	CG31649 - CG10833		2y(15)	CG16869 - CG31649	
			2m(18)	CG16869 - CG16870		2y(18)	CG10833 - CG16870	
								
2L(3)			2m(15)	CG9159 - CG9162		2y(14)	CG9162 - CG5203	
			2m(17)	CG5198 - CG5203		2y(17)	CG5198 - CG9159	
								
2LR(4)			2m(19)	CG15274 - CG4182		2y(19) ^1^	CG15274 - CG9397	
			2m(25)	CG12110 - CG9397		2y(21) ^1^	CG4182 - CG12110	
								
2LR(5)			2m(20)	CG5818 - CG4440		2y(20) ^1^	CG11777 - CG5818	
			2m(26)	CG11777 - CG12891		2y(23) ^1^	CG12891 - CG4440	
								
2L(6)			2m(21)	CG4580 - CG4599		2y(24) ^1^	CG4580 - CG15152	
			2m(22)	CG15152 - CG5758		2y(25)	CG4599 - CG5758	
								
2LR(7)			2m(23)	CG2614 - CG31678		2y(26) ^1^	CG2614 - CG8118	
			2m(28)	CG8118 - CG30483		2y(28) ^1^	CG31678 - CG30483	
								
2LR(8)			2m(24)	CG31674 - CG9333		2y(22) ^1^	CG9333 - CG12388	
			2m(27)	CG12388 - CG12387		2y(27) ^1^	CG12387 - CG31674	
								
2R(9)			2m(29)	CG16801 - CG8166		2y(29)	CG16801 - CG13504	
			2m(32)	CG13504 - CG6741		2y(32)	CG8166 - CG6741	
								
2R(10)			2m(30)	CG6518 - CG6622		2y(31)	CG30264 - CG6518	
			2m(34)	CG30264 - CG30272		2y(34)	CG6622 - CG30272	
								
2R(11)			2m(31)	CG4847 - CG4853		2y(30)	CG4853 - CG10384	
			2m(33)	CG10972 - CG10384		2y(33)	CG10972 - CG4847	
								
3L(1)			3m(35)	CG32305 - CG1275		3y(35) ^1^	CG32305 - CG8189	
			3m(38)	CG8189 - CG6749		3y(38) ^1^	CG1275 - CG6749	
								
3L(2)			3m(36)	CG32280 - CG32278		3y(36) ^1^	CG7999 - CG32278	
			3m(37)	CG7986 - CG7999		3y(37) ^1^	CG7986 - CG32280	
								
3L(3), 3L(4)			3m(39)	CG32147 - CG6859		3y(39) ^1^	CG32147 - CG3979	
			3m(41)	CG4561 - CG33158		3y(40) ^1^	CG33158 - CG6859	
			3m(42)	CG3979 - CG6865		3y(42) ^1^	CG6865 - CG4561	
								
3L(5)			3m(40)	CG5215 - CG5444		3y(41) ^1^	CG5215 - CG7405	
			3m(43)	CG7405 - CG7407		3y(43) ^1^	CG5444 - CG7407	
								
3R(6)			3m(44)	CG2519 - CG10390		3y(44)	CG2519 - CG14332	
			3m(52)	CG33333 - CG14332		3y(52)	CG33333 - CG10390	
								
3R(7), 3R(8)			3m(45)	CG31286 - CG1315		3y(45)	CG31176 - CG2708	
			3m(46)	CG2708 - CG7918		3y(46) ^1^	CG1315 - CG7918	
			3m(53)	CG31176 - CG5849		3y(53) ^1^	CG31286 - CG5849	
								
3R(9)			3m(47)	CG14708 - CG10898		3y(47) ^1^	CG14708 - CG31266	
			3m(51)	CG31266 - CG31267		3y(51) ^1^	CG10898 - CG31267	
								
3R(10), 3R(11)			3m(48)	CG31211 - CG31366		3y(48) ^1^	CG14900 - CG31366	
			3m(49)	CG3028 - CG3041		3y(49) ^1^	CG3028 - CG14899	
			3m(50)	CG14899 - CG14900		3y(50) ^1^	CG3041 - CG31211	
								
3R(12)			3m(54)	CG31142 - CG5289		3y(54)	CG31142 - CG11504	
			3m(55)	CG11504 - CG7908		3y(55)	CG5289 - CG7908	
^1^ Verified by sequencing (see Material and Methods).
