# Supplementary material for: Principles of Genome Evolution in the Drosophila melanogaster Species Group
Source: PLoS Biol. 2007 Jun 5;5(6):e152. doi: 10.1371/journal.pbio.0050152 (PMC1885836; doi:10.1371/journal.pbio.0050152)
Supplement: Supplementary file 9 [file pbio.0050152.st002.rtf]

Table S2.  Size of syntenic blocks between D. melanogaster and D. yakuba with the number of genes that have, or have not, been mapped between them.	
			Size (kb)		Genes	
Chromosome	Rank	Reference Genes	D. melanogaster	D. yakuba		Mapped	Non-Mapped	
X	1	Telomere X - CG14804 ^2^	1281.64	1374.474		161	15	
X	2	CG14805 - CG12729	1732.637	1614.989		361	21	
X	3	CG15764 - CG5921	3957.868	3789.101		25	0	
X	4	CG5928 - CG14434	195.197	190.725		88	3	
X	5	CG32737 - CG2194	784.664	743.418		225	10	
X	6	CG12119 - CG2186	2334.215	2374.562		148	10	
X	7	CG2157 - CG1697 ^2^	1657.458	1626.169		88	5	
X	8	CG2247 - CG1502 ^2^	729.23	678.081		47	0	
X	9	CG18130 - CG32659	378.81	353.129		20	4	
X	10	CG2577 - CG13012	342.055	330.655		433	40	
X	11	CG13010 - CG14195	4297.746	4124.439		258	12	
X	12	CG7874 - CG9578 ^2^	2532.92	2517.685		103	8	
X	13	CG9579 - Centromere ^1,2^	958.018	855.455		136	11	
2	14	Telomere 2L - CG3036	4903.889	4913.901		546	15	
2	15	CG2837 - CG31649	301.322	284.895		49	0	
2	16	CG10833 - CG9159	863.484	869.613		115	1	
2	17	CG9162 - CG7233	1897.738	1919.25		228	2	
2	18	CG7231 - CG5198	2453.399	2498.169		357	9	
2	19	CG5203 - CG16869	3474.09	3473.47		383	6	
2	20	CG16870 - CG15274	1115.92	1145.554		81	10	
2	21	CG4182 - CG5818	1293.658	1314.29		107	1	
2	22	CG4440 - CG4580	160.189	163.032		14	0	
2	23	CG4599 - CG15152	1623.48	1613.437		114	4	
2	24 	CG5758 - CG2614 ^2^	2530.858	2373.238		254	7	
2	25 	CG31678 - CG31674 ^2^	173.654	161.015		23	1	
2	26 	CG9333 - CG12110 ^1,2^	3350.038	3911.186		149	35	
2	27	CG9397 – CG11777	3967.392	3823.79		580	11	
2	28	CG12891 – CG12388	873.306	863.181		100	2	
2	29	CG12387 – CG11777	2719.712	2704.4		357	6	
2	30	CG30483 - CG16801	1276.798	1249.073		155	9	
2	31	CG8166 - CG6518	1551.425	1560.591		205	5	
2	32	CG6622 - CG4847	588.906	570.629		76	2	
2	33	CG4853 - CG13504	4609.483	4625.378		573	15	
2	34	CG6741 - CG10972	292.596	294.352		46	0	
2	35	CG10384 - CG30264	362.393	357.164		56	1	
2	36	CG30272 - Telomere 2R	2429.489	2455.175		401	12	
3	37	Telomere 3L - CG32305	2236.261	2205.577		264	7	
3	38	CG1275 - CG32280	916.319	914.393		93	2	
3	39	CG32278 - CG7986	5011.844	5055.751		571	10	
3	40	CG7999 - CG8189	1539.953	1503.544		229	3	
3	41	CG6749 - CG32147	5404.1	5544.024		529	17	
3	42	CG6859 - CG5215	968.045	1026.947		56	51	
3	43	CG5444 - CG4561 ^2^	306.032	259.494		35	42	
3	44	CG33158 - CG3979	2426.467	2396.367		230	7	
3	45	CG6865 - CG7405	2927.743	3100.658		242	150	
3	46	CG7407 - CG2519 ^1,2^	3388.082	3906.967		211	40	
3	47	CG10390 - CG31286	1095.024	1031.022		152	3	
3	48 	CG1315 - CG2708 ^2^	1394.25	1546.676		120	32	
3	49	CG7918 - CG14708	3495.523	3404.86		432	6	
3	50	CG10898 - CG31211	396.43	398.285		57	0	
3	51	CG31366 - CG3028	2006.312	2002.966		255	7	
3	52	CG3041 - CG14899	2519.487	2534.261		313	3	
3	53	CG14900 - CG31266	649.016	657.202		71	1	
3	54	CG31267 - CG33333	283.711	291.174		27	0	
3	55	CG14332 - CG31176	4297.857	4593.274		401	55	
3	56	CG5849 - CG31142	2189.886	2257.683		283	6	
3	57	CG5289 - CG11504	6069.93	6097.866		763	15	
3	58	CG7908 - Telomere 3R	2078.778	2082.77		217	4	
4	59 	CG17923 – CG18026 ^1^	3495.523	3404.86		75	5	
^1^ Syntenic blocks containing a centromere; their sizes are an underestimate since most part of the centromeric heterochromatin in them has not considered.
^2^ Syntenic block for which the coefficient of variation of its size is above 5%.
