# Supplementary material for: Principles of Genome Evolution in the Drosophila melanogaster Species Group
Source: PLoS Biol. 2007 Jun 5;5(6):e152. doi: 10.1371/journal.pbio.0050152 (PMC1885836; doi:10.1371/journal.pbio.0050152)
Supplement: Supplementary file 10 [file pbio.0050152.st003.rtf]

TABLE S3.  Conservative transposition events detected between D. melanogaster and D. yakuba.	
	D. melanogaster		D. yakuba	
Gene (x)	Region 1	Region 2		Region 1	Region 2	
CG13962	2L: CG10528-x-CG13958	2L: CG10443-CG10462		2R: CG10528-CG13958	2R: CG10443-x-CG10462	
CG9068	2R: CG30324-x-CG7755	2L: CG8902-CG11289		2R: CG30324-CG7755	2L: CG8902-x-CG11289
