# Supplementary material for: Principles of Genome Evolution in the Drosophila melanogaster Species Group
Source: PLoS Biol. 2007 Jun 5;5(6):e152. doi: 10.1371/journal.pbio.0050152 (PMC1885836; doi:10.1371/journal.pbio.0050152)
Supplement: Supplementary file 11 [file pbio.0050152.st004.rtf]

TABLE S4.  Duplicative transposition events detected between D. melanogaster and D. yakuba.	
		Targeted Region	
Potential Gene Source	Potential Derivatives (x)	D. melanogaster	D. yakuba	
X: CG1740	CG10174*	2L: CG10302-x-CG10283	-	
X: CG32584	CG13732*	3L: CG6333-x-CG13733	-	
	CG13732a	-	3R: CG4316-x-CG4001	
X: CG12359	CG11023	2R: CG12343-x-CG12938	-	
X: CG12655	CG32511	X: CG1819-x-CG14579	-	
X: CG32708	CG10993	X: CG33174-x-CG10996	-	
X: CG32678	CG32671/CG32670	X: Nested into CG2174	-	
	CG9807	X: CG15295-x-CG9806	-	
	CG2885	X: CG32683-x-CG12640	-	
	CG32673	X: CG2111-x-CG12637	-	
X: CG14213	CG9573	2L: CG9568-x-CG9582	-	
X: CG2033	CG12324*	2R: CG122938-x-CG12343	-	
2L: CG8137	CG9334	2L: CG9333-x-CG31676	-	
2L: CG10595-CG13088	(CG10595-CG13088)a	-	2R: CG1219-x-CG18445	
2L: CG18241	CG18241a	-	2L: CG9289-x-CG9287	
	CG18241b	-	2L: Nested into CG31762	
2L: CR33315	CG15172	2L: CG15171-x-CG10446	-	
2R: CG4866	CG4866a	-	2R: CG10972-x-CG4847	
2R: CG10327	CG7804	3L: CG33259-x-CG7815	-	
2R: CG3584	CG9337	2L: CG9336-x-CG9338	-	
	CG4021	2R: CG30395-x-CG4402	-	
	CG3875	2R: CG3746-x-CG4373	-	
	CG33317	2L: CG9339-x-CG9342	-	
	CG3927	2R: CG12489-x-CG4269	-	
3L: CG7692	CG9902	X: CG9903-x-CG9901	-	
3R: CG33213	CG33221	X: CG32788-x-CG32789	-	
3R: CG9821	CG32797	X: CG12496-x-CG7952	-	
	CG32733	X: CG4575-x-CG32732	-	
3R: CG11958	CG1924	X: CG2750-x-CG15733	-	
	CG9906 (nested into CG9907)	X: CG4429-x-CG9903	-	
* Previously reported [1].
Duplicates of D. melanogaster genes in D. yakuba have been suffixed 'a' and 'b'.


1. Betran E, Thornton K, Long M (2002) Retroposed new genes out of the X in Drosophila. Genome Res 12: 1854-1859.
