# Supplementary material for: Principles of Genome Evolution in the Drosophila melanogaster Species Group
Source: PLoS Biol. 2007 Jun 5;5(6):e152. doi: 10.1371/journal.pbio.0050152 (PMC1885836; doi:10.1371/journal.pbio.0050152)
Supplement: Supplementary file 12 [file pbio.0050152.st006.rtf]

TABLE S6.  Repeat composition characterization by RepeatMasker in co-occurrent breakpoint regions of D. melanogaster and D. yakuba.	
	Type of Mobile Element	
Junction	LINE	LTR	DNA	
D. melanogaster	
Xm(1)	0	0	0	
Xm(9)	0	1	0	
				
Xm(2)	0	0	1	
Xm(4)	0	0	0	
				
Xm(3)	0	0	0	
Xm(8)	0	0	0	
				
Xm(5)	0	0	0	
Xm(11)	0	0	0	
				
Xm(6)	0	0	0	
Xm(12)	0	0	0	
				
Xm(7)	0	0	0	
Xm(10)	0	0	0	
				
2m(13)	0	0	0	
2m(16)	0	3	0	
				
2m(14)	0	0	1	
2m(18)	0	0	0	
				
2m(15)	0	0	0	
2m(17)	0	0	1	
				
2m(19)	0	0	0	
2m(25)	45	94	39	
				
2m(20)	0	0	0	
2m(26)	0	0	0	
				
2m(21)	0	0	0	
2m(22)	0	0	0	
				
2m(23)	0	0	0	
2m(28)	0	0	0	
				
2m(24)	0	0	0	
2m(27)	0	0	0	
				
2m(29)	0	3	0	
2m(32)	0	0	0	
				
2m(30)	0	0	0	
2m(34)	0	0	0	
				
2m(31)	0	0	0	
2m(33)	1	0	0	
				
3m(35)	0	0	0	
3m(38)	0	0	0	
				
3m(36)	0	0	0	
3m(37)	0	0	0	
				
3m(39)	0	0	0	
3m(41)	0	0	0	
3m(42)	0	0	2	
				
3m(40)	0	0	0	
3m(43)	0	0	0	
				
3m(44)	0	0	0	
3m(52)	0	0	0	
				
3m(45)	0	0	0	
3m(46)	3	0	0	
3m(53)	0	0	0	
				
3m(47)	0	1	0	
3m(51)	0	0	0	
				
3m(48)	0	0	0	
3m(49)	0	0	0	
3m(50)	0	0	0	
				
3m(54)	0	0	0	
3m(55)	0	0	0	
				
Total	3	5	5	
				
D. yakuba	
Xy(1)	0	0	0	
Xy(4)	0	2	1	
				
Xy(3)	0	0	0	
Xy(8)	0	4	3	
				
Xy(2)	0	0	0	
Xy(9)	0	0	0	
				
Xy(7)	0	0	0	
Xy(11)	0	0	0	
				
Xy(6)	0	0	0	
Xy(12)	4	0	0	
 				
Xy(5)	0	0	0	
Xy(10)	0	0	0	
				
2y(13)	0	0	0	
2y(16)	0	0	0	
				
2y(15)	0	0	0	
2y(18)	0	0	5	
				
2y(14)	0	0	1	
2y(17)	0	0	0	
				
2y(19)	0	0	0	
2y(21)	6	1	10	
				
2y(20) ^1^	0	0	1	
2y(23) ^1^	1	0	3	
				
2y(24) ^1^	0	3	12	
2y(25) ^1^	17	2	2	
				
2y(26)	0	0	0	
2y(28)	0	0	0	
				
2y(22) ^1^	1	0	0	
2y(27) ^1^	0	0	1	
				
2y(29)	0	0	0	
2y(32)	0	0	0	
				
2y(31)	0	0	0	
2y(34)	0	0	5	
				
2y(30)	0	5	0	
2y(33)	0	0	0	
				
3y(35)	0	0	0	
3y(38)	0	0	0	
				
3y(36)	0	0	0	
3y(37)	0	0	0	
				
3y(39) ^1^	0	0	1	
3y(40)	0	0	0	
3y(42) ^1^	0	0	7	
				
3y(41)	0	0	0	
3y(43)	0	0	1	
				
3y(44) ^1^	0	0	1	
3y(52) ^1^	1	0	3	
				
3y(45)	1	0	6	
3y(46)	0	0	6	
3y(53)	0	0	0	
				
3y(47)	0	2	0	
3y(51)	1	0	3	
				
3y(48)	0	0	5	
3y(49)	0	0	0	
3y(50)	0	0	1	
				
3y(54)	0	0	0	
3y(55)	0	0	0	
				
Total	8	7	21	
^1^ Co-occurrent breakpoint regions with identical repetitive sequence.
