# Supplementary material for: Principles of Genome Evolution in the Drosophila melanogaster Species Group
Source: PLoS Biol. 2007 Jun 5;5(6):e152. doi: 10.1371/journal.pbio.0050152 (PMC1885836; doi:10.1371/journal.pbio.0050152)
Supplement: Supplementary file 13 [file pbio.0050152.st007.rtf]

TABLE S7.  Primers used in this work for cloning and/or sequencing.	
Code ^1^	Sequence	
Xy(9) f	AGCATCCGTCGTGGAACCAT	
Xy(9) r	TCACGCGGAAAAGTCCATCA	
Xy(10) f	ACCGTCAAGTCCGCCAAGTC	
Xy(10) r	TGCCAGGGCATGAAGACTGA	
2y(19) f	CGCGGACTACAACATCTGATACAAT	
2y(19) r	ACACATGGCGAAGAACATCTCTAGT	
2y(20) f	TAATCTTGCGGCAGAATCAG	
2y(20) r	TGGCGACGTGATAGAGTGC	
2y(21)A f	GTCAACCTGCCATTGGACTT	
2y(21)A r	CGGATGCTCGGATCTACACT	
2y(21)B f	TGGAAGAGGCATGATGGACT	
2y(21)B r	ACGTACGCGATGTCACCTTC	
2y(21)C f	GATCATTTGCAAAGAAAGACACACC	
2y(21)C r	CGAATCCGAGAGTTGGCTTAAAT	
2y(21)D f	TAATTAGGCGGAAACTCCGATTCTA	
2y(21)D r	GGGAAACTTACCTTGGCACAGTGA	
2y(22) f	CACTCGTGCTTGTAGCTGGA	
2y(22) r	CGAGCAGATGGCTGAGAAC	
2y(23) f	TGCTGCTGTCCGAAGGAAC	
2y(23) r	TTGACCGACATTCGTAGCAT	
2y(24) f	TTGCTCCGGTCTTCCAGTC	
2y(24) r	TGCTGGAACTCGCCTATTCT	
2y(26) f	ACTAGCGGCCAATCACTACG	
2y(26) r	GAATGAGTTGTCCGCCTCAA	
2y(27) f	CCGCCTCGAATCAACTACTC	
2y(27) r	CTTCGCGTAATGTCCTTCAA	
2y(28) f	ACCTCATCCACCACCAAGAA	
2y(28) r	TGCCACTCACAACGAATAGC	
3y(35) f	GAAGAAGACGAGGCTGGAGA	
3y(35) r	TCGAATCTGAATGGAAGGAG	
3y(36) f	GTCGGCGATGTAATGGAACT	
3y(36) r	TGAGGAGGAACTTCTTGACCA	
3y(37) f	TGCATGGATGTCCATCACTC	
3y(37) r	TAACGACGGTGGCTCTATGG	
3y(38) f	TCAATCTCGATGTGCTGGAG	
3y(38) r	GGTTGTCTACCTGGCCACTG	
3y(39) f	CGGTGTCACTTCCGAAGATT	
3y(39) r	ACAGCCGCCACAATCTTATC	
3y(40) f	GGATACTCGGATCTGGTTCG	
3y(40) r	TGCTGCTGCTCTTCATGGTA	
3y(41) f	GCGTAGGTGGCATTGGTTAC	
3y(41) r	ACATTGCAGACGTGAGTTCG	
3y(42)A f	AGATGCGCAACTAATTTGAGACTCC	
3y(42)A r	TCTGCATTCTTCGTCTCAACCTTC	
3y(42)B f	TGGCATCTTGCCAGCTAATTACAA	
3y(42)B r	CCAACTATGGGAATACCTCCAGAGC	
3y(42)C f	GCGCCTTAGGTACTTTAGGGTTACA	
3y(42)C r	GGGTGTACTCCTTGGACAGCTGATA	
3y(43) f	CGTTCGTGTCTATGCGTCTG	
3y(43) r	AACCTTCGTCCTCCAGTTCC	
3y(46)1 f	ACAAGCAGTATGAGGTCGTATGTGTCC	
3y(46)1 r	AATTGCGCTTACAGCCAACACTAAG	
3y(46)2 f	ACTTAACCTACGGCTCATATTGTGGCTA	
3y(46)2 r	TTCATCAAGCTGCCATCTCTTATCG	
3y(46)3 f	CTGCTCACCACAATCATCTTGTGTA	
3y(46)3 r	TTGACAGTTATCTCTCTGGCCAACAA	
3y(46)A f	ATGAAGGCATGCACCATGTA	
3y(46)A r	TATTTCAGACCCGAGGGCTA	
3y(46)B 	CAACCCAGGGAAACTGAAGA	
3y(46)C f	TTGGCTGTAAGCGCAATTTA	
3y(46)C r	GAATTCGCTGGGACTCACAT	
3y(46)D f	CCCAGCGAATTCAACAGAATA	
3y(46)D r	CAAACACAAGGAGCCGTACA	
3y(46)E f	ACATCAAGAACTGCGGAACC	
3y(46)E r	AATTTCGTGCCGAGTTTACG	
3y(46)F f	TGATGTGTCAAGCGTTCGAT	
3y(46)F r	CAACATACCCGCACTGCTAA	
3y(46)G	CACGCGTCAGGGTCAGAATG	
3y(46)H	CCTATCCTCCTGGGCTCCAAA	
3y(46)I	TGGAGCCCAGGAGGATAGGG	
3y(46)J	CGGCCGTCTTGACACAAATG	
3y(46)K	CGCATGAGGATTGCTGTTCG	
3y(46)L	CGGACAGTGACCAACTGACTGA	
3y(46)M	CGGAGCGTATGCGTAACGAG	
3y(46)N	GCAATGACCGCACATCGAAA	
3y(46)O	CTCGCTCCGTGTGTTTGCAC	
3y(46)P	GGCCGATAAGTGTGTGCAGTTC	
3y(46)Q	CCGTGTCCGGACCTGTAAGTT	
3y(46)R	TTGCCAGCGAGAAGATGTCG	
3y(47)A f	ACAGAGGCCATGGGCACCTA	
3y(47)A r	GCCGTCGATGGGAAGATTTG	
3y(47)B f	AGCTGCGAGTCCCAATCCTG	
3y(47)B r	AAACCCCGTGGTGTTGGATG	
3y(48)A f	CCTCTCCACCGCAGTCACCT	
3y(48)A r	CCGCGAGCTGTACGAGACCT	
3y(48)B f	CCAGCAGGCAGGAGGATTTG	
3y(48)B r	CCGCGCGTAACTTTTCCTTG	
3y(49) f	TTGGCCCAGGAACAACAGGT	
3y(49) r	GGCGTCTACGAACGGGACAC	
3y(50) f	AATCCAGATCCGCCCAGTGA	
3y(50) r	GGTGCCGGCCACATTGTATT	
3y(51) f	CGGATGCAGATGAGGGCTGT	
3y(51) r	CGCCAGGACATCGGACTCTT	
3y(53)A f	CATCGATACTTCGGGATTTCA	
3y(53)A r	ATGTTAGCGTCGCCATAACC	
3y(53)B f	AGACCCGAGGGCTAAAGATT	
3y(53)B r	ACCGCGAAAGCATCACTTA	
3y(53)C f	TGGGCCCTCTGAATTATTTG	
3y(53)C r	GTGCACATTCCCGGAGTATC	
3y(53)E	CGCGGTCCAAAGCAAACTCT	
3y(53)F	GCTCAGGGTGCGGCCTTTAT	
3y(53)H	TGCAATTGGGAAATCCATGC	
Sim a f	GTCGGTCAAGTGCTGTGAGA	
Sim a r	ATCGCGAGGTAAGGAATGC	
Sim b f	CTGACCAGCGACTTGGTCTT	
Sim b r	GAGACGGAGATGGAGATGGA	
Sim c f	GATTGCTGCTCGACGATGT	
Sim c r	TCGGCACTAACGACTTGTCA	
Sim d f	AGGATGCTAAGCTGCCACTG	
Sim d r	TTGATCCAACTGCAGCACTC	
Sim e f	GGAGCCACCATGACTTAACG	
Sim e r	GAAGGTCCTGAAGAACTCACCT	
CG2708 f	GTCTTTGCAAGCTGGGTAGC	
CG2708 r	CGTTATTGCCCTCGAACATT	
CG7918 f	CCTGAACCAGAGCCTCAGTC	
CG7918 r	GGCGTAGCAGAGGAAGTACG	
CG31176 f	CTCGAAGCCCATTCTGAGTC	
CG31176 r	TTCGGGAGGACTTGTAGGTG	
CG34034 f	gctgagcaatgtggatgct	
CG34034 r	cctggttgagaggattaagctg	
CG5289 f	CGTTTACCCAGCGATCAGTT	
CG5289 r	ACCTTGTAGTTGCGCTCGAT	
CG6576 f	ATGTTCGCAAGACCGTGAA	
CG6576 r	GCTGAGAACGCTTGAATTGG	
HDC14860 f	ataacagaggcggcaacg	
HDC14860 r	aagtcgctgccaagtgattc	
HDC14861 f	ccaccaaacaagtgctgct	
HDC14861 r	tatttggagtcgggccacat	
HDC12400 f	ccatccaacattctccgact	
HDC12400 r	atccactggcaccaatatcc	
HDC14862 f	TATTCTGGGCATTGTGGTTG	
HDC14862 r	ACTGCGCTGAGCCAAACTAA	
pdf800 f	cgcaagtagaaagacgatgct	
pdf800 r	gataaaaggagcatgccaaa	
^1^ Relative to the targeted region.
